# Supplementary material for: Statin therapy inhibits fatty acid synthase via dynamic protein modifications
Source: Nat Commun. 2022 May 10;13:2542. doi: 10.1038/s41467-022-30060-w (PMC9090928; doi:10.1038/s41467-022-30060-w)
Supplement: Supplementary file 1 — Supplementary Information [file 41467_2022_30060_MOESM1_ESM.pdf]

Supplementary Information: Statin therapy inhibits fatty acid synthase via dynamic protein modifications

MD Hirschey et al.

a

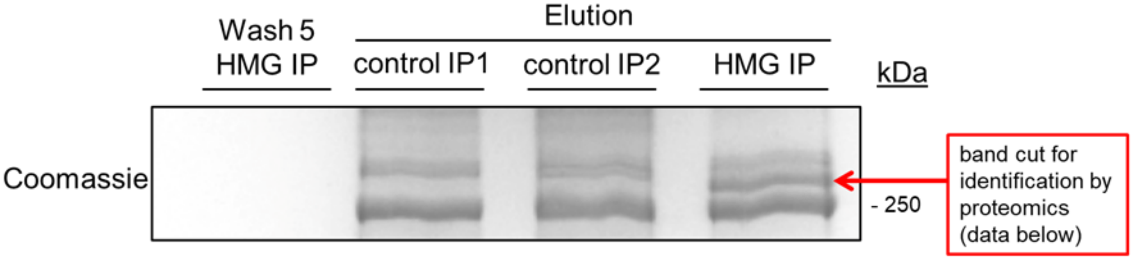

b

Protein	Total Peptides	Peptide Ratio (HMG IP/control IP)	Molecular Weight (kDa)
**Fatty acid synthase**	**895**	**6.22 (895/144)**	**273**
Spectrin beta chain, non-erythrocytic 1	406	1.24 (406/328)	275
Myosin-10	187	0.79 (187/238)	229
Keratin, type II cytoskeletal 1	148	1.66 (148/89)	66
Spectrin alpha chain, non-erythrocytic 1	91	1.25 (91/73)	285

Supplementary Fig. 1: Immunoprecipitation of HMGylated protein in statin-treated cells

The HMGylated protein from statin-treated HepG2 cells was identified as fatty acid synthase via an IP followed by mass spectrometry. (**a**) Coomassie gel showing the proteins separated from the HMG IP versus the control IPs utilizing a pre-immune serum. The unique band occurring above 250 kDa was cut out of the gel for subsequent mass spectrometry. The corresponding area in the control IP was cut out as a baseline for mass spectrometry results. (Single experiment). (**b**) A table showing the proteins detected in each band. FAS was present in a higher ratio in the HMG IP indicating it is the HMGylated protein. Source data are provided as a Source Data file.

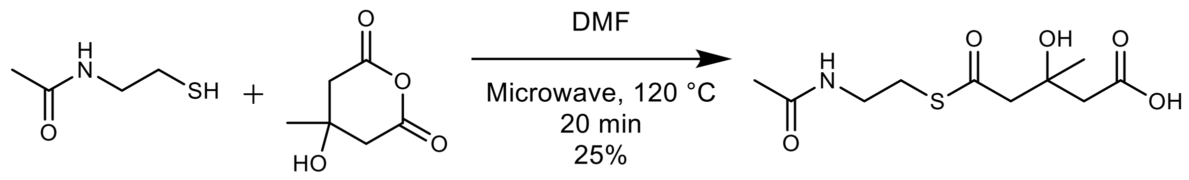

Supplementary Fig. 2: 5-(2-acetamidoethylthio)-3-hydroxy-3-methylglutaric acid (HMG-NAC)

Chemical synthesis and structure of HMG-NAC. HMG-NAC is used as an HMG anhydride generating reagent to compare non-enzymic transfer of HMG to protein vs transfer that require the CoA handle to facilitate transfer. Briefly, N-acetylcysteamine (34 µL, 0.32 mmol) and 3-hydroxy-3-methylglutaric anhydride (50 mg, 0.35 mmol) were dissolved in *N,N*-dimethylformamide (DMF, 2 mL) and subjected to microwave irradiation in a 2-5 mL Biotage microwave vial at 120 °C for 20 minutes. The reaction mixture was concentrated under reduced pressure and was purified by flash chromatography (0→40% CH_3OH:CH_2Cl_2) to yield the product as white solid (21 mg, 25%). Chemicals: N-acetylcysteamine (Sigma #363340), 3-hydroxy-3-methylglutaric anhydride (Cayman Chemical #18632)

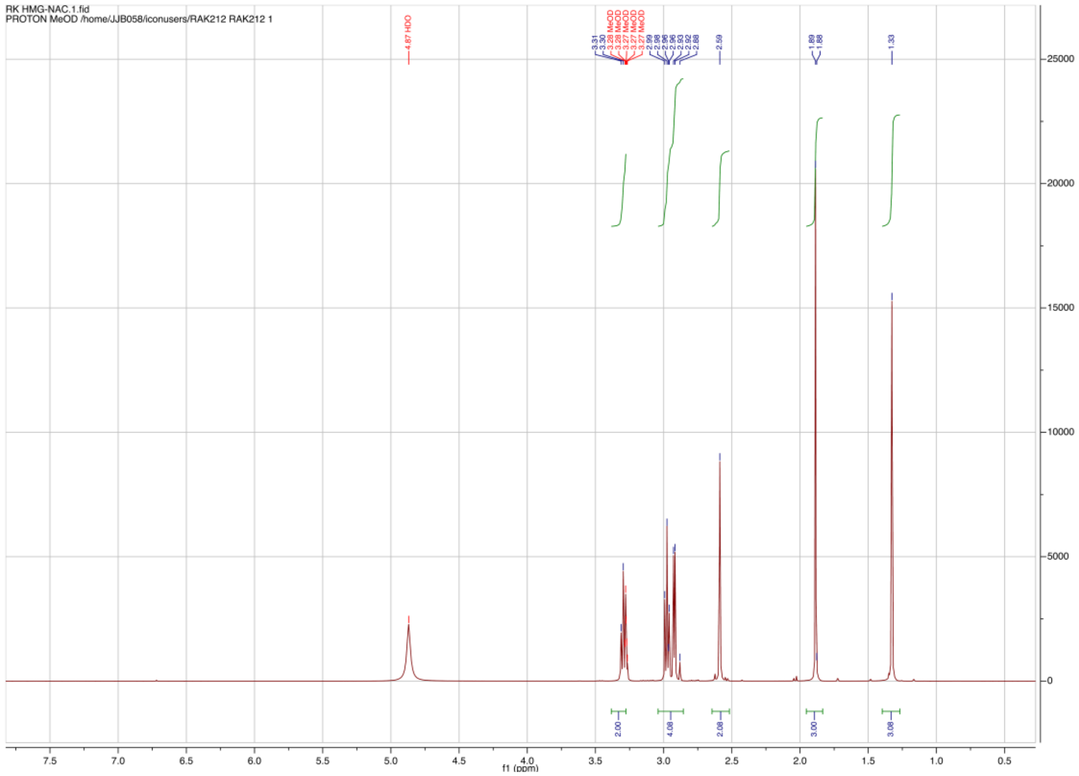

Supplementary Fig. 3. ^{1}H- spectra for HMG-NAC ^{1}H NMR (400 MHz, CD_3OD) δ 3.31-3.27 (m, 2H), 2.99-2.88 (m, 4H), 2.63-2.54 (m, 2H), 1.89 (s, 3H), 1.22 (s, 3H).

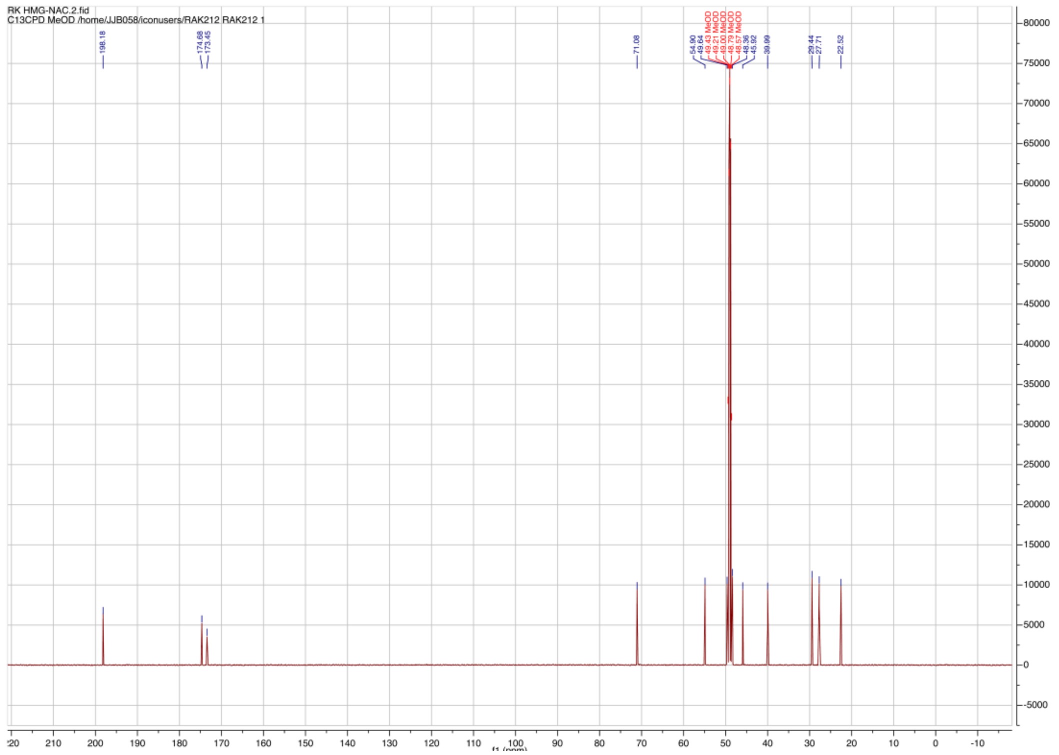

Supplementary Fig. 4. ^{13}C-NMR spectra for HMG-NAC. ^{13}C NMR (100 MHz, CD_3OD) δ 198.18, 174.68, 173.45, 71.08, 54.90, 45.92, 39.99, 29.44, 27.71, 22.52. MS (ESI+): 264.1 $(M+H^+)$, (ESI-): 262.1 $(M-H^+)$.

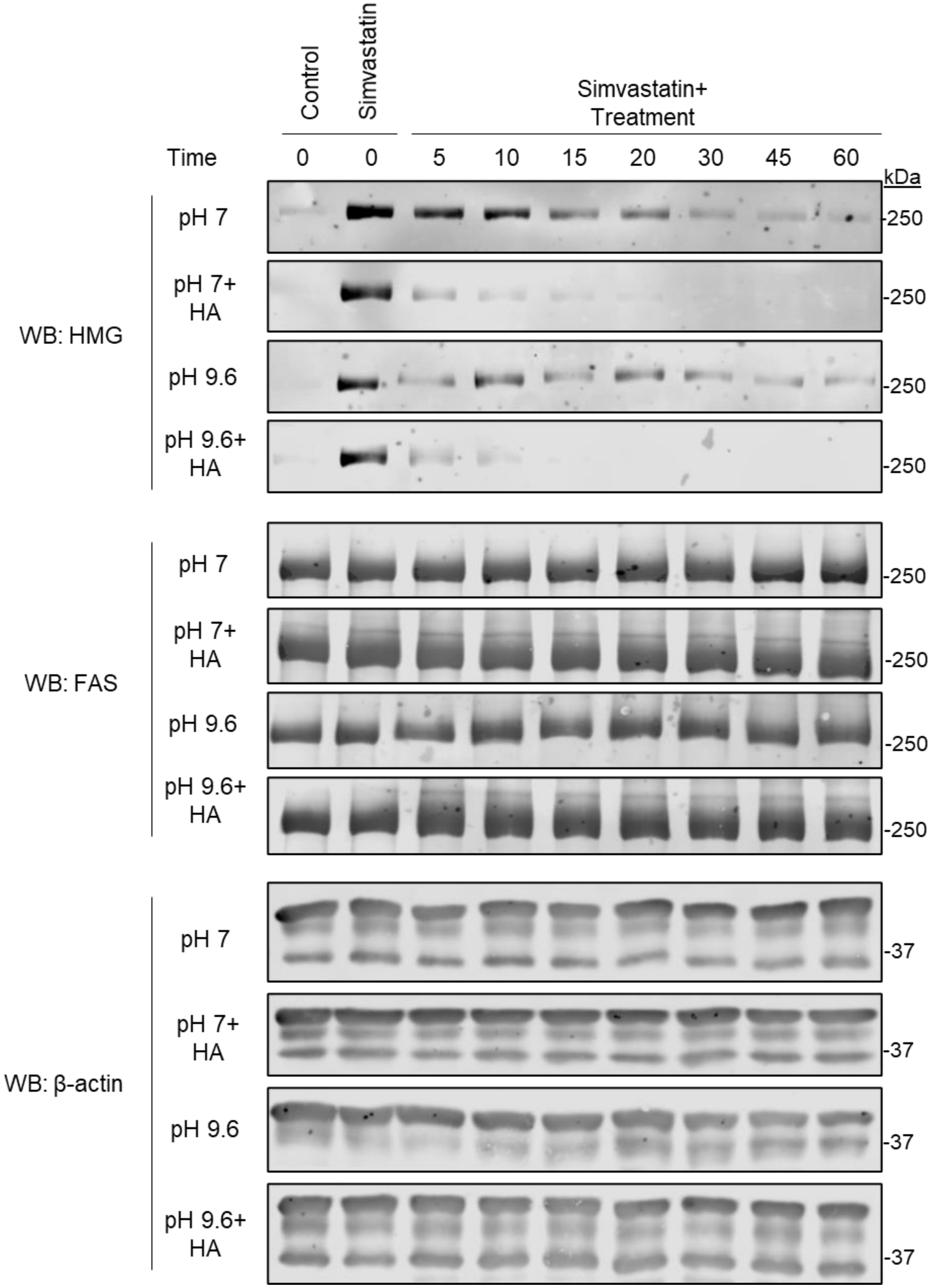

Control
Simvastatin
Simvastatin+ Treatment
Time 0 0 5 10 15 20 30 45 60
kDa
WB: HMG
pH 7
pH 7+ HA
pH 9.6
pH 9.6+ HA
-250
WB: FAS
pH 7
pH 7+ HA
pH 9.6
pH 9.6+ HA
-250
WB: β-actin
pH 7
pH 7+ HA
pH 9.6
pH 9.6+ HA
-37

Supplementary Fig. 5: Loading controls for HMGylated FAS treated with hydroxylamine

Treatment of lysates containing HMGylated FAS with hydroxylamine at pH 7 or 9.6 show the HMG modification is labile and therefore a thioester or ester bond. Expanded image of Figure 3F including the loading controls that show FAS levels remain constant while the HMGylation signal diminishes with treatment (representative of 3 independent experiments). Source data are provided as a Source Data file.

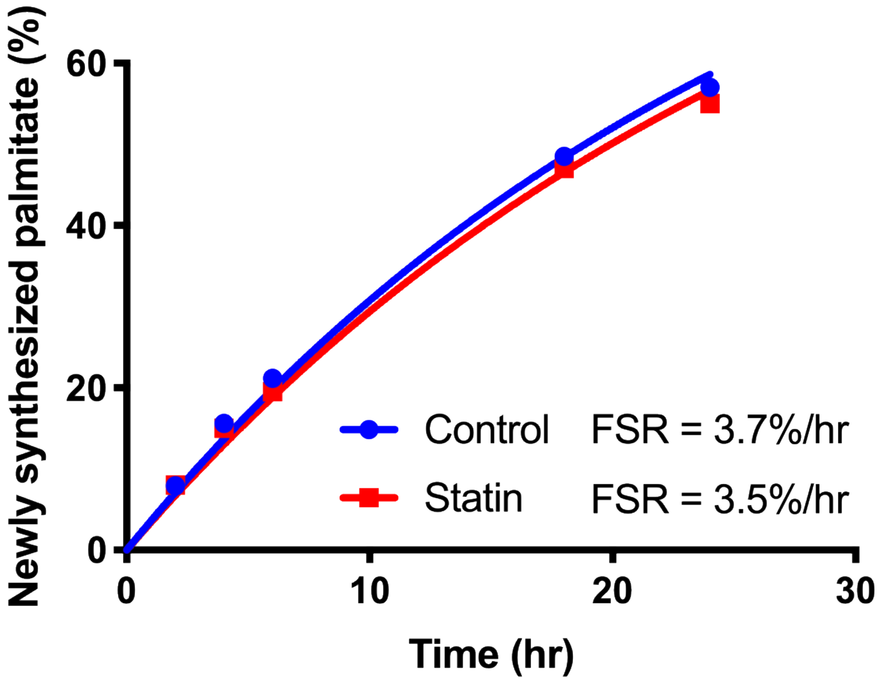

Supplementary Fig. 6: Fractional Synthesis Rate of Palmitate in control and statin-treated cells HepG2

The fraction of newly synthesized palmitate was fitted vs time to determine the fractional synthesis rate (%/hr). The fractional synthesis rates between control and statin-treated HepG2 cells were not significantly different. n = 3 biological replicates. Source data are provided as a Source Data file.

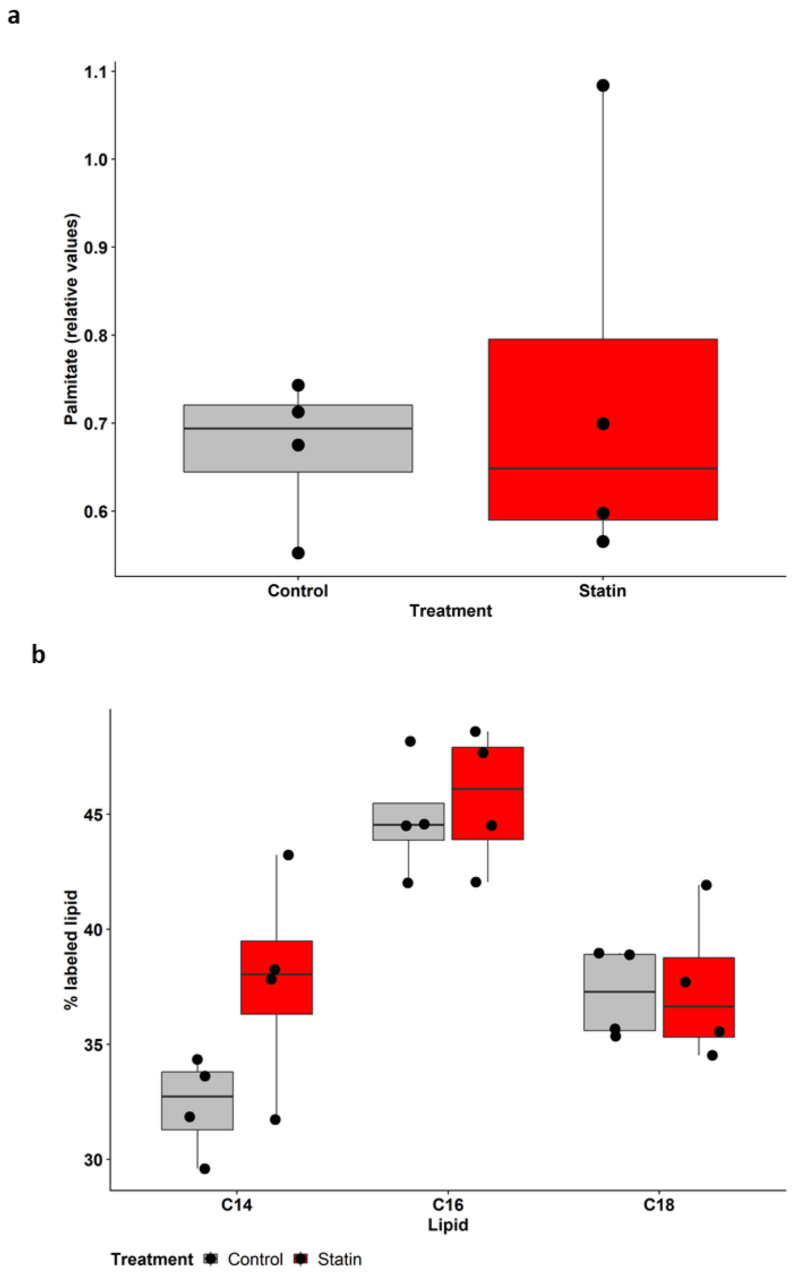

Supplementary Fig. 7: Measurement of lipid in control and statin-treated mice

Analysis of hepatic lipids from control and statin-treated mice. (**a**) Total palmitate levels in the liver were determined by normalization to an added control lipid (C17). No change in total palmitate levels was detected between treatment groups ($p = 0.6$, $n = 4$ biological replicates, ANOVA). (**b**) Levels of less common FAS products, myrisate (C14) and stearate (C18), were analyzed using the same labeled tissues. No changes were detected in newly synthesized myristate ($p = 0.8$, $n = 4$ biological replicates, ANOVA) or stearate ($p = 0.9$, $n = 4$ biological replicates, ANOVA). Raw data is shown as dots in addition to a boxplot generated from a best fit model, where the box represents the first to third quartiles and “whiskers” represent the data extending beyond this range. Outliers were determined by being more than 1.5 IQR from the 25 percentile. Source data are provided as a Source Data file.

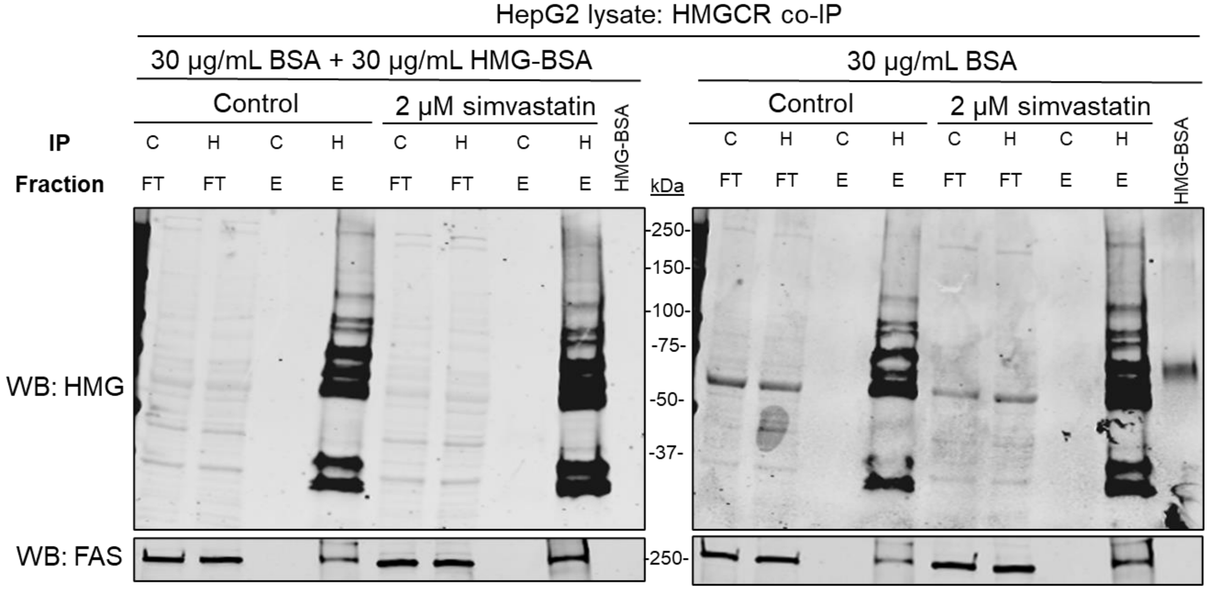

Supplementary Fig. 8: FAS co-precipitates with HMGCR and is HMGylated.

A co-IP reveals that FAS co-precipitates with HMGCR regardless of statin treatment and that during statin treatment this portion of FAS is HMGylated. The blots on the left are competition blots where HMGylated BSA is added to the antibody stock to bind any HMG sensing antibody, while the right blot allows for antibodies to bind HMGylation sites. The statin treated eluate shows an HMGylation signal at ~250 kDa that disappears during antibody competition, indicating the signal is HMGylation of FAS. HMG-BSA was run as a control to show the competition blot was effective. Representative of three independent experiments. *C = Control IP with protein A only; H = HMGCR IP; FT = flow through fraction from IP; E = eluate from IP. Source data are provided as a Source Data file.

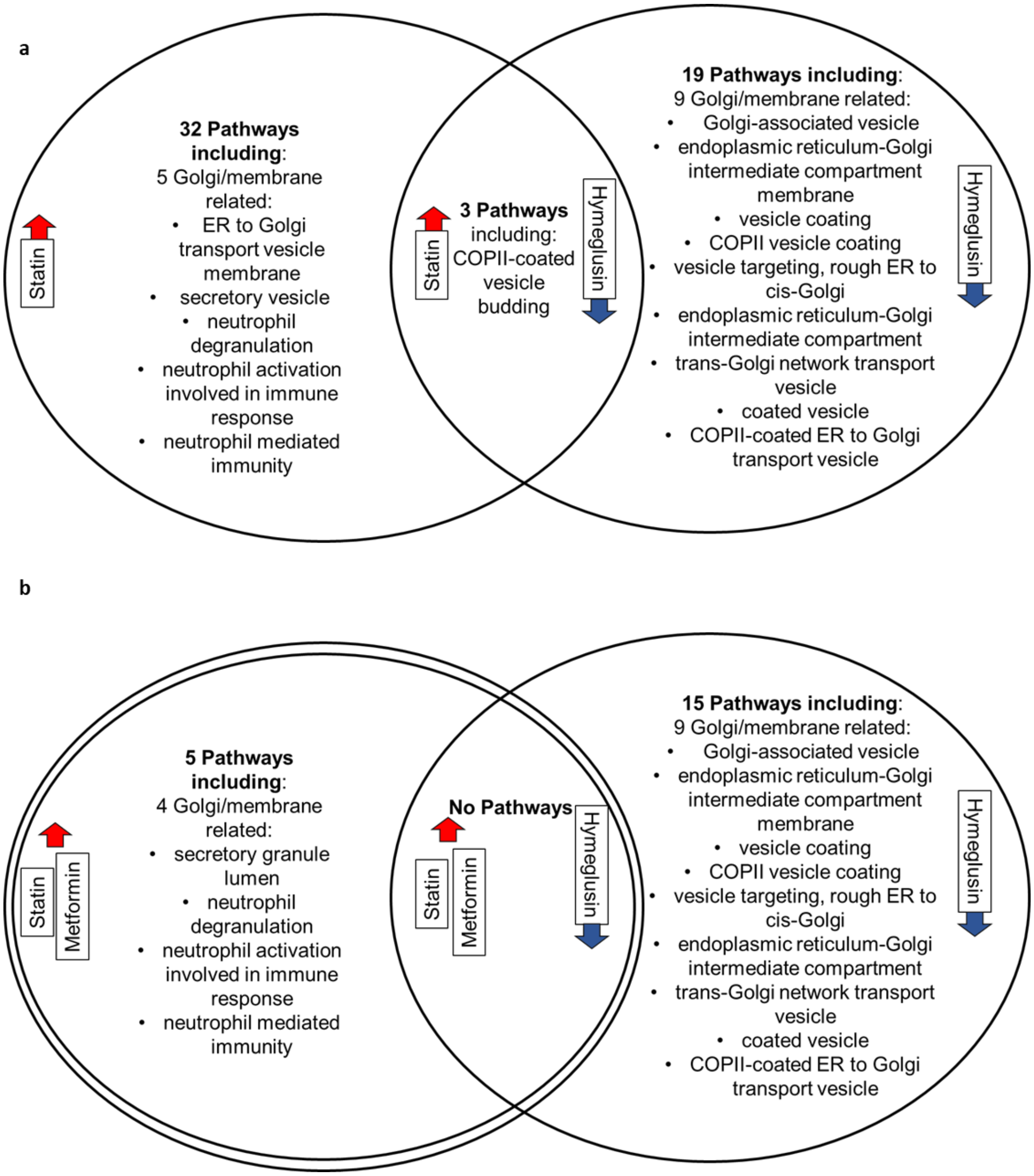

Supplementary Fig. 9: Venn Diagram breakdown of treatment intersections of enriched pathways from the label-free quantitative proteomics experiment.

Venn Diagrams compliment the display of data from the upsetter plots in Figures 6 and 7. (a) Venn Diagram corresponding to Upsetter plot from Figure 6f. Shows the GO pathways that are increasing during statin treatment, decreasing during hymeglusin treatment, or fit both categories. (b) Venn Diagram corresponding to Upsetter plot from Figure 7c. Includes data from metformin treated cells that overlap with statin treatment so all pathways shown change in the same direction during both statin and metformin treatment. Shows the GO pathways that are increasing during statin and metformin treatment, decreasing during hymeglusin treatment, or fit both categories. Source data are provided as a Source Data file.

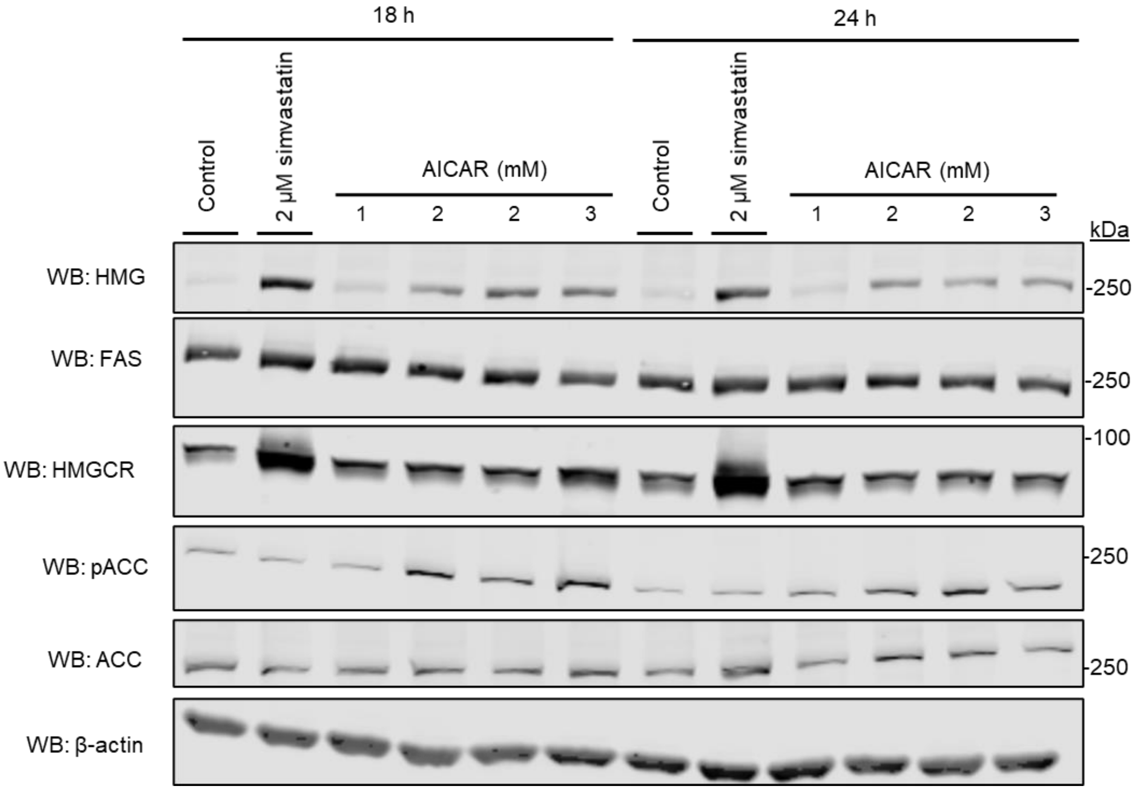

Supplementary Fig. 10: AMPK activation with AICAR results in HMGylation of FAS

HepG2 cells were treated with the AMPK activator AICAR for 18 or 24 hours. Western blotting reveals a singular HMGylation band consistent with the results observed in metformin- or statin-treated cells. Phosphorylated ACC shows AMPK is activated during HMGylation of FAS. Representative of 2 independent experiments. Source data are provided as a Source Data file.
