# Supplementary material for: Statin therapy inhibits fatty acid synthase via dynamic protein modifications
Source: Nat Commun. 2022 May 10;13:2542. doi: 10.1038/s41467-022-30060-w (PMC9090928; doi:10.1038/s41467-022-30060-w)
Supplement: Supplementary file 3 — Reporting Summary [file 41467_2022_30060_MOESM3_ESM.pdf]

nature portfolio

Corresponding author(s): Matthew Hirschey
Last updated by author(s): Jan 14, 2022

Reporting Summary

Nature Portfolio wishes to improve the reproducibility of the work that we publish. This form provides structure for consistency and transparency in reporting. For further information on Nature Portfolio policies, see our Editorial Policies and the Editorial Policy Checklist.

Statistics

For all statistical analyses, confirm that the following items are present in the figure legend, table legend, main text, or Methods section.

n/a	Confirmed	
☐	☒	The exact sample size (*n*) for each experimental group/condition, given as a discrete number and unit of measurement
☐	☒	A statement on whether measurements were taken from distinct samples or whether the same sample was measured repeatedly
☐	☒	The statistical test(s) used AND whether they are one- or two-sided<br>*Only common tests should be described solely by name; describe more complex techniques in the Methods section.*
☒	☐	A description of all covariates tested
☒	☐	A description of any assumptions or corrections, such as tests of normality and adjustment for multiple comparisons
☐	☒	A full description of the statistical parameters including central tendency (e.g. means) or other basic estimates (e.g. regression coefficient) AND variation (e.g. standard deviation) or associated estimates of uncertainty (e.g. confidence intervals)
☐	☒	For null hypothesis testing, the test statistic (e.g. *F*, *t*, *r*) with confidence intervals, effect sizes, degrees of freedom and *P* value noted<br>*Give P values as exact values whenever suitable.*
☒	☐	For Bayesian analysis, information on the choice of priors and Markov chain Monte Carlo settings
☒	☐	For hierarchical and complex designs, identification of the appropriate level for tests and full reporting of outcomes
☒	☐	Estimates of effect sizes (e.g. Cohen's *d*, Pearson's *r*), indicating how they were calculated

Our web collection on statistics for biologists contains articles on many of the points above.

Software and code

Policy information about availability of computer code

Data collection	No software was used for data collection.
Data analysis	R (4.0) was used for data analysis, and all code is freely available on the github repository located at github.com/hirscheylab. Additionally, proteomic data analysis was performed using Proteome Discoverer 2.2.

For manuscripts utilizing custom algorithms or software that are central to the research but not yet described in published literature, software must be made available to editors and reviewers. We strongly encourage code deposition in a community repository (e.g. GitHub). See the Nature Portfolio guidelines for submitting code & software for further information.

Data

Policy information about availability of data

All manuscripts must include a data availability statement. This statement should provide the following information, where applicable:

- Accession codes, unique identifiers, or web links for publicly available datasets
- A description of any restrictions on data availability
- For clinical datasets or third party data, please ensure that the statement adheres to our policy

Source data are provided with this paper. Raw LC-MS/MS data used in figures 4, 5 and 6 have been deposited to the ProteomeXchange Consortium via the PRIDE partner repository with the dataset identifier PXD030952. Proteomic datasets are available as supplemental tables. All source data files are available at the persistent digital object identifier 10.5281/zenodo.5819042. PDB entry for molecular visualization is 6YNV.

Field-specific reporting

Please select the one below that is the best fit for your research. If you are not sure, read the appropriate sections before making your selection.

[x] Life sciences [ ] Behavioural & social sciences [ ] Ecological, evolutionary & environmental sciences

For a reference copy of the document with all sections, see nature.com/documents/nr-reporting-summary-flat.pdf

Life sciences study design

All studies must disclose on these points even when the disclosure is negative.

Sample size	No sample size calculation was performed in this study. Instead, sample sizes were chosen based on previous measurements and standard practices.
Data exclusions	No data were excluded from the analyses.
Replication	At least three independent replicates were performed. Exact number of replicates for each experiment are indicated in the methods and figure legends
Randomization	Studies using cells and mice were randomized for treatment and control.
Blinding	No samples were blinded prior to analysis.

Reporting for specific materials, systems and methods

We require information from authors about some types of materials, experimental systems and methods used in many studies. Here, indicate whether each material, system or method listed is relevant to your study. If you are not sure if a list item applies to your research, read the appropriate section before selecting a response.

Materials & experimental systems

n/a	Involved in the study	
[ ]	[x]	Antibodies
[ ]	[x]	Eukaryotic cell lines
[x]	[ ]	Palaeontology and archaeology
[ ]	[x]	Animals and other organisms
[x]	[ ]	Human research participants
[x]	[ ]	Clinical data
[x]	[ ]	Dual use research of concern

Methods

n/a	Involved in the study	
[x]	[ ]	ChIP-seq
[x]	[ ]	Flow cytometry
[x]	[ ]	MRI-based neuroimaging

Antibodies

Antibodies used	Antibodies and ratios used were HMG at 1:1000 (Millipore ABS2108), FAS at 1:5000 (abcam ab184619), HMGCR at 1:1000 (Millipore ABS229), Phosphorylated ACC at 1:1000 (CST 3661), total ACC (CST 3662), FAS monoclonal at (Sigma-Aldrich, WH0002194M1). Secondary antibodies were LI-COR 926-32213 donkey against rabbit or LI-COR 925-68072 donkey against mouse and used at 1:10000. PLA probes were made using Sigma Duolink Probemaker kit. HMGCR antibody from abcam (ab214018) was ligated to PLUS oligo and FAS antibody from abcam (ab18461) was ligated to MINUS oligo following Probemaker instructions.
Validation	Validation was performed by matching the pattern with suppliers product information, molecular weights, and replication across experimental conditions and systems.

Eukaryotic cell lines

Policy information about cell lines

Cell line source(s)	HepG2 cells were obtained from ATCC
Authentication	Authentication confirmed by ATCC
Mycoplasma contamination	n/a
Commonly misidentified lines (See ICLAC register)	No commonly misidentified cell lines used in this study

Animals and other organisms

Policy information about studies involving animals; ARRIVE guidelines recommended for reporting animal research

Laboratory animals	C57/Bl6NJ Mice were obtained from Jackson Labs and received ad libitum PicoLab rodent diet 20 (#5053, LabDiet, St. Louis, MO) and water. About 2-4 animals per cage were housed together with a 12 hr light-dark cycle. Both male and female mice were used in experiments at 12-24 weeks of age to account for potential differences due to sex and litter mates used in experiments to control for age differences.
Wild animals	No wild animals were used in this study.
Field-collected samples	No field-collected samples were used in this study.
Ethics oversight	All animal procedures were performed in accordance with the Association for the Assessment and Accreditation of Laboratory Animal Care (AAALAC) international guidelines and approved by the Duke University Institutional Animal Care & Use Committee under protocol number A065-20-03.

Note that full information on the approval of the study protocol must also be provided in the manuscript.
