# Supplementary material for: Statin therapy inhibits fatty acid synthase via dynamic protein modifications
Source: Nat Commun. 2022 May 10;13:2542. doi: 10.1038/s41467-022-30060-w (PMC9090928; doi:10.1038/s41467-022-30060-w)
Supplement: Supplementary file 4 — Source Data [file 41467_2022_30060_MOESM4_ESM.zip › source_data/Supplementary Fig/VennDiagrams.pptx]

## Slide 1
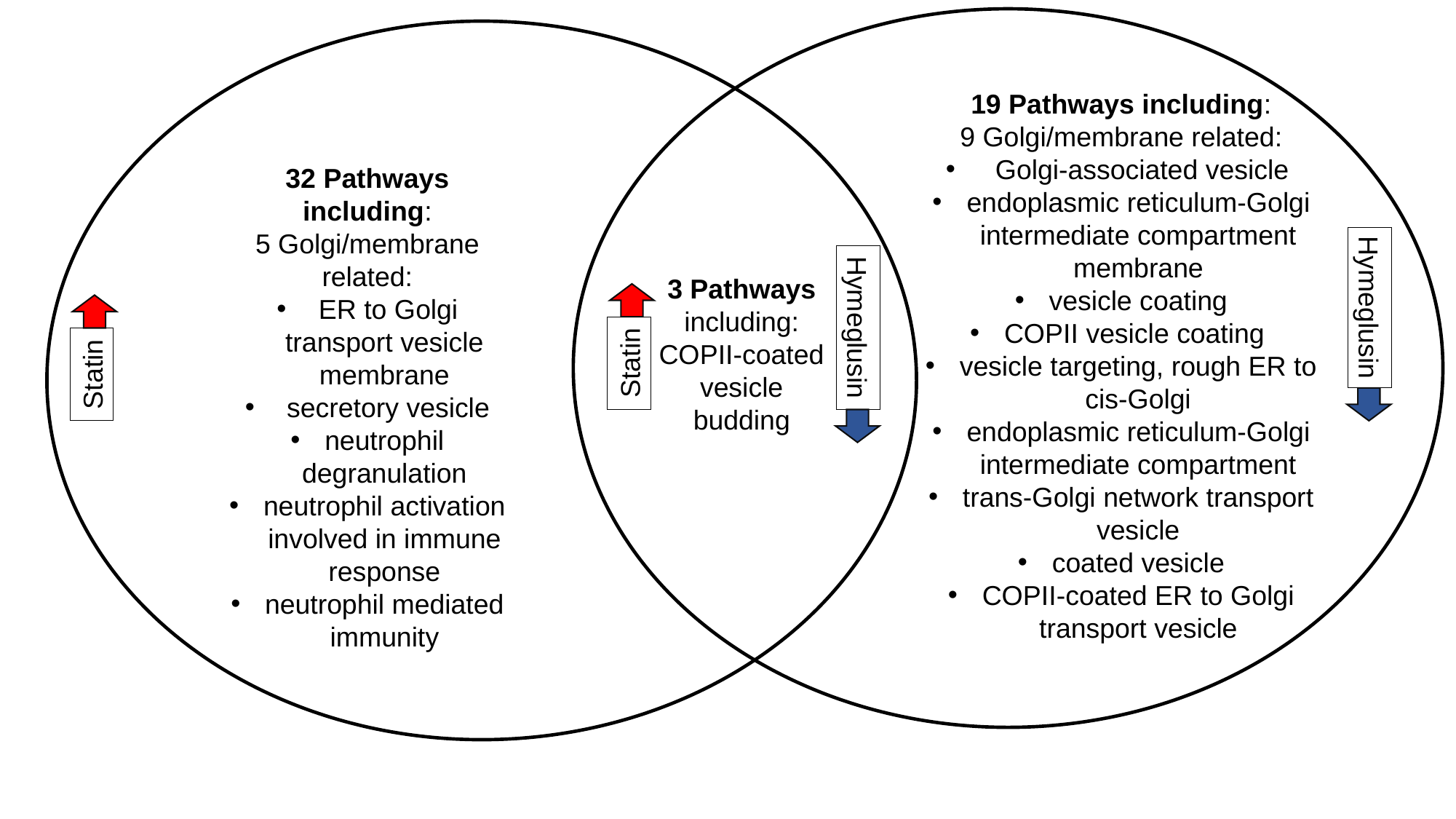

19 Pathways including:
9 Golgi/membrane related:
 Golgi-associated vesicle
endoplasmic reticulum-Golgi intermediate compartment membrane
vesicle coating
COPII vesicle coating
vesicle targeting, rough ER to cis-Golgi
endoplasmic reticulum-Golgi intermediate compartment
trans-Golgi network transport vesicle
coated vesicle
COPII-coated ER to Golgi transport vesicle
32 Pathways including:
5 Golgi/membrane related:
 ER to Golgi transport vesicle membrane
 secretory vesicle
neutrophil degranulation
neutrophil activation involved in immune response
neutrophil mediated immunity
3 Pathways including:
COPII-coated vesicle budding
Hymeglusin
Hymeglusin
Statin
Statin

## Slide 2
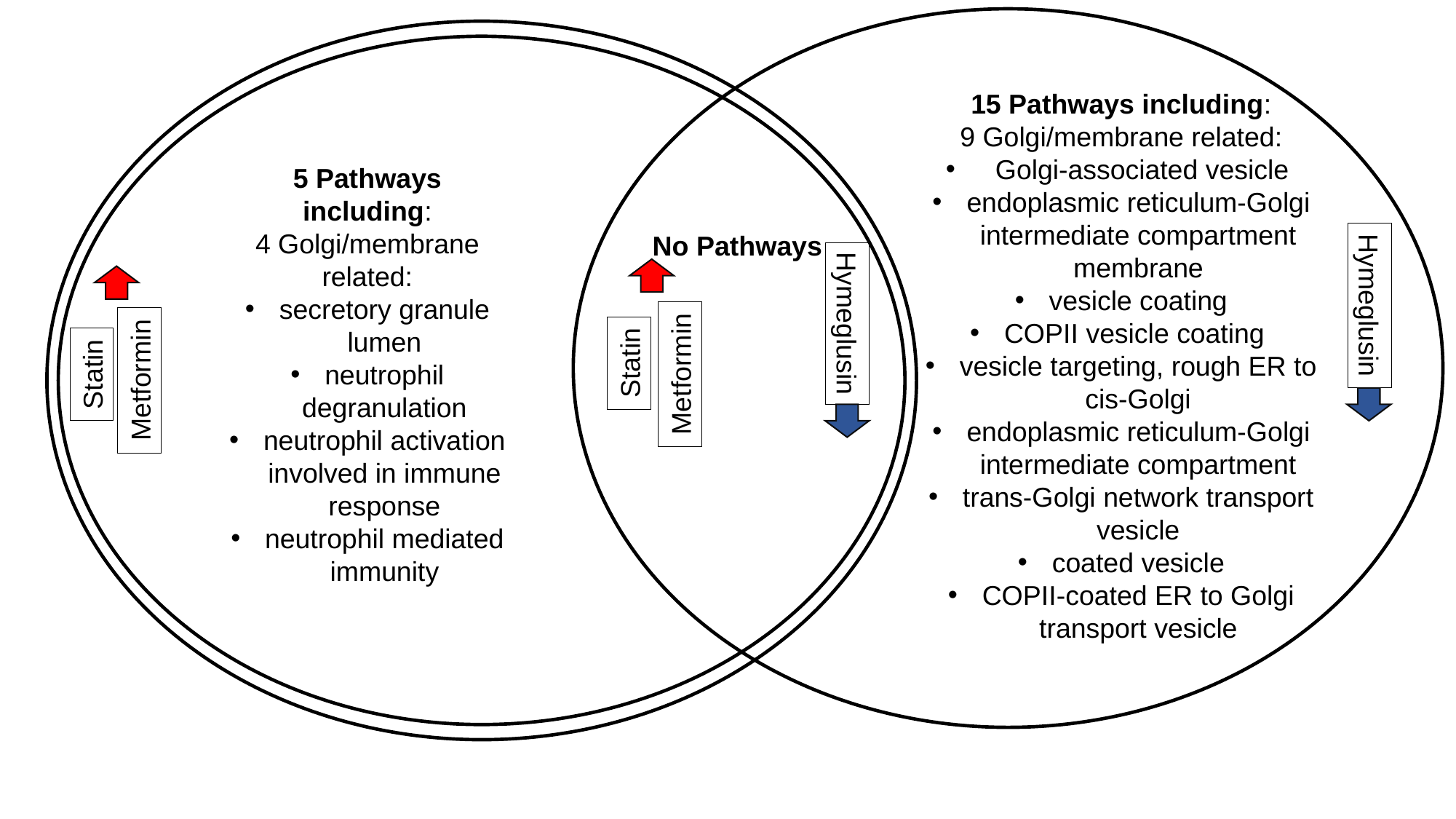

15 Pathways including:
9 Golgi/membrane related:
 Golgi-associated vesicle
endoplasmic reticulum-Golgi intermediate compartment membrane
vesicle coating
COPII vesicle coating
vesicle targeting, rough ER to cis-Golgi
endoplasmic reticulum-Golgi intermediate compartment
trans-Golgi network transport vesicle
coated vesicle
COPII-coated ER to Golgi transport vesicle
5 Pathways including:
4 Golgi/membrane related:
secretory granule lumen
neutrophil degranulation
neutrophil activation involved in immune response
neutrophil mediated immunity
No Pathways
Hymeglusin
Hymeglusin
Statin
Statin
Metformin
Metformin
