# Supplementary material for: Statin therapy inhibits fatty acid synthase via dynamic protein modifications
Source: Nat Commun. 2022 May 10;13:2542. doi: 10.1038/s41467-022-30060-w (PMC9090928; doi:10.1038/s41467-022-30060-w)
Supplement: Supplementary file 4 — Source Data [file 41467_2022_30060_MOESM4_ESM.zip › source_data/Figure 4/Western blot info.docx]

Protein Markers:

Bio Rad Precision Plus Protein Dual Color Standards #1610374:

Top to Bottom (kDa): 250, 150, 100, 75, 50, 37, 20, 15, 10

Figure 4a:

- FAS blot
  - Markers 250 to 75
  - abcam ab184619 diluted 1:5000
- HMG blot
  - Millipore ABS2108 1:1000
  - Markers 250 to 75

Figure 4b:

- FAS blot
  - abcam ab184619 diluted 1:5000
- HMG blot
  - Millipore ABS2108 1:1000
  - Markers 250 to 100

Figure 4c:

- FAS blot
  - Markers 250 to 75
  - abcam ab184619 diluted 1:5000
- HMG blot
  - Millipore ABS2108 1:1000
  - Markers 250 to 75
- Beta actin blot
  - Markers 50, 38, compressed band at bottom is bottom of gel
- HMGCR
  - Millipore ABS229
  - Markers 250 to 75

Figure 4d (left side of raw blots):

- FAS blot
  - Markers 250 to 75 are visible
  - abcam ab184619 diluted 1:5000
- HMG blot
  - Millipore ABS2108 1:1000
  - Markers 250 to 38
- HMGCR
  - Millipore ABS229
  - Markers 250 to 38

Figure 4e (right side of raw blots):

- FAS blot
  - Markers 250 to 75 (visible only on left side)
  - abcam ab184619 diluted 1:5000
- HMG blot
  - Millipore ABS2108 1:1000
  - Markers 250 to 75
- Beta actin blot
  - Markers 75, 50, 38

Figure 4f:

- FAS blot
  - Ph7: Markers 250 to 75 (top blot), 250, 150 bottom blot
  - Ph9.6: Markers 250 to 75
  - abcam ab184619 diluted 1:5000
- HMG blot
  - Millipore ABS2108 1:1000
  - Markers 250 to 75
- Beta actin blot
  - Markers 75, 50, 38
