# Supplementary figures and images for: Statin therapy inhibits fatty acid synthase via dynamic protein modifications
Source: Nat Commun. 2022 May 10;13:2542. doi: 10.1038/s41467-022-30060-w (PMC9090928; doi:10.1038/s41467-022-30060-w)

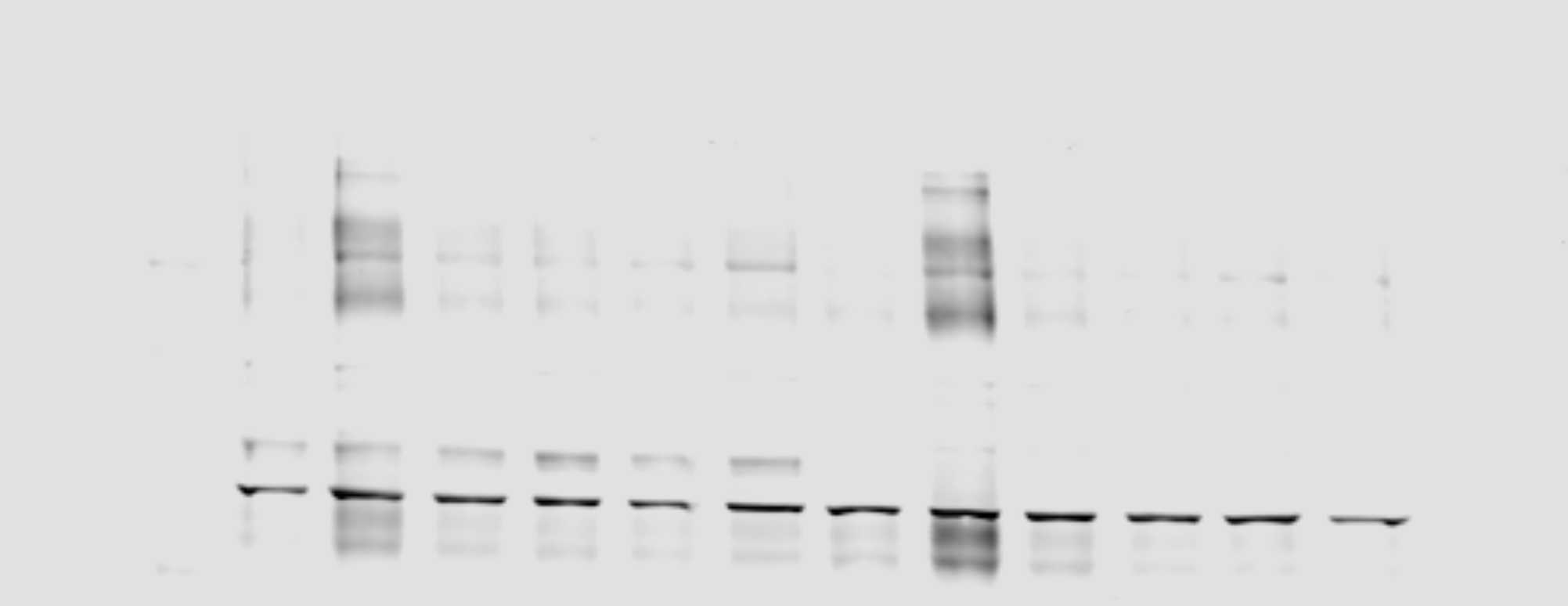

Supplement: Supplementary file 4 — Source Data [file 41467_2022_30060_MOESM4_ESM.zip › source_data/Figure 7/Fig7b_2017-10-11_Metformin_HMGCR.tif]

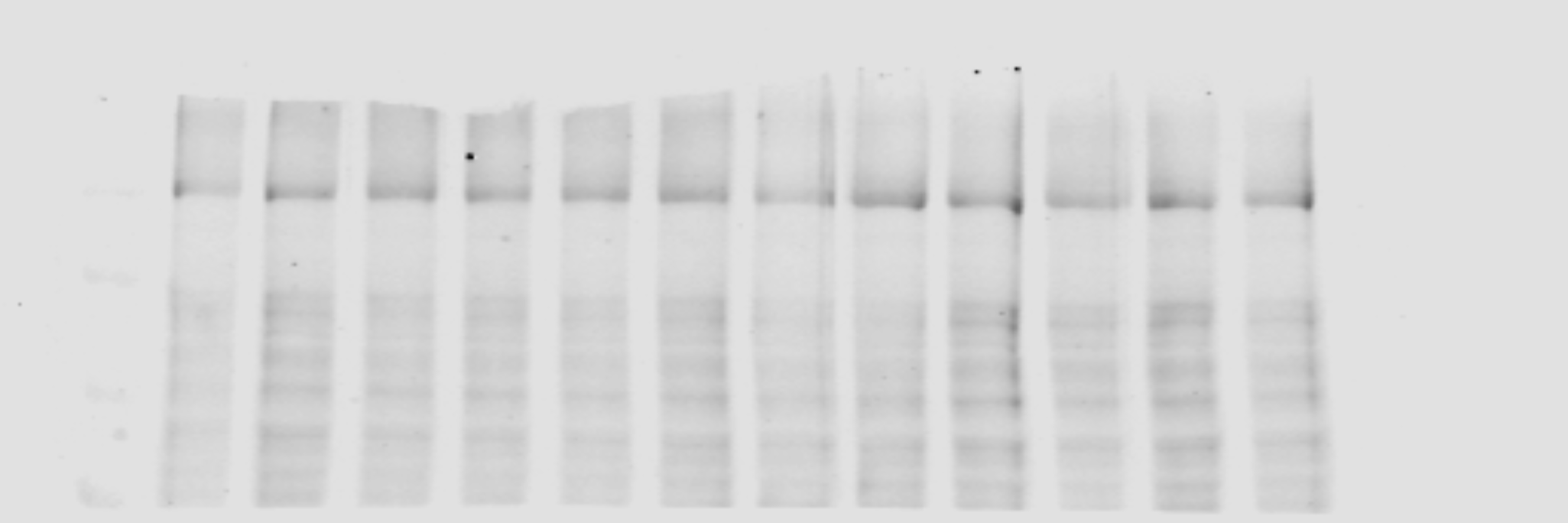

Supplement: Supplementary file 4 — Source Data [file 41467_2022_30060_MOESM4_ESM.zip › source_data/Figure 7/Fig7b_2017-10-10_Metformin_ACC.tif]

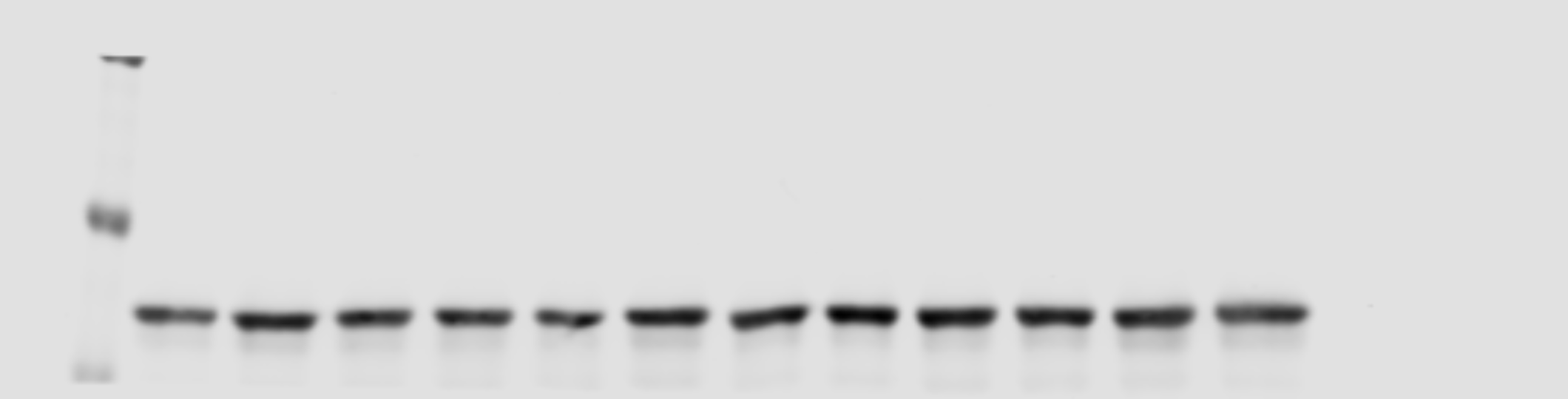

Supplement: Supplementary file 4 — Source Data [file 41467_2022_30060_MOESM4_ESM.zip › source_data/Figure 7/Fig7b_2017-10-10_Metformin_bactin for pACC.tif]

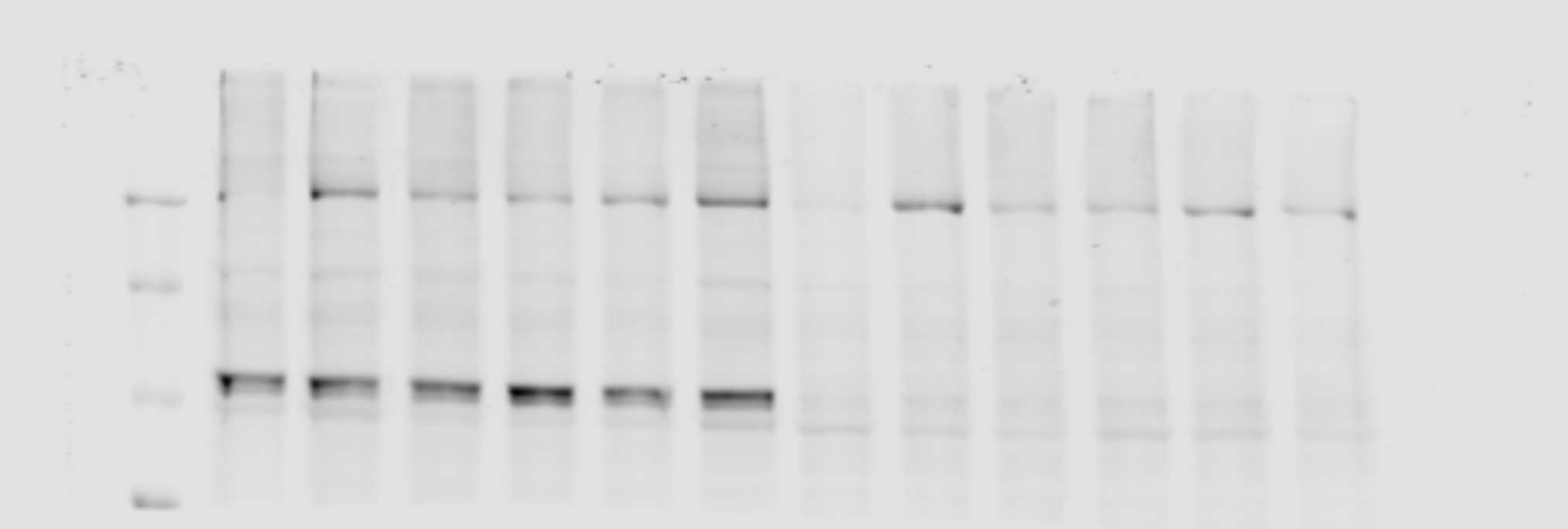

Supplement: Supplementary file 4 — Source Data [file 41467_2022_30060_MOESM4_ESM.zip › source_data/Figure 7/Fig7b_2017-10-10_Metformin_HMG.tif]

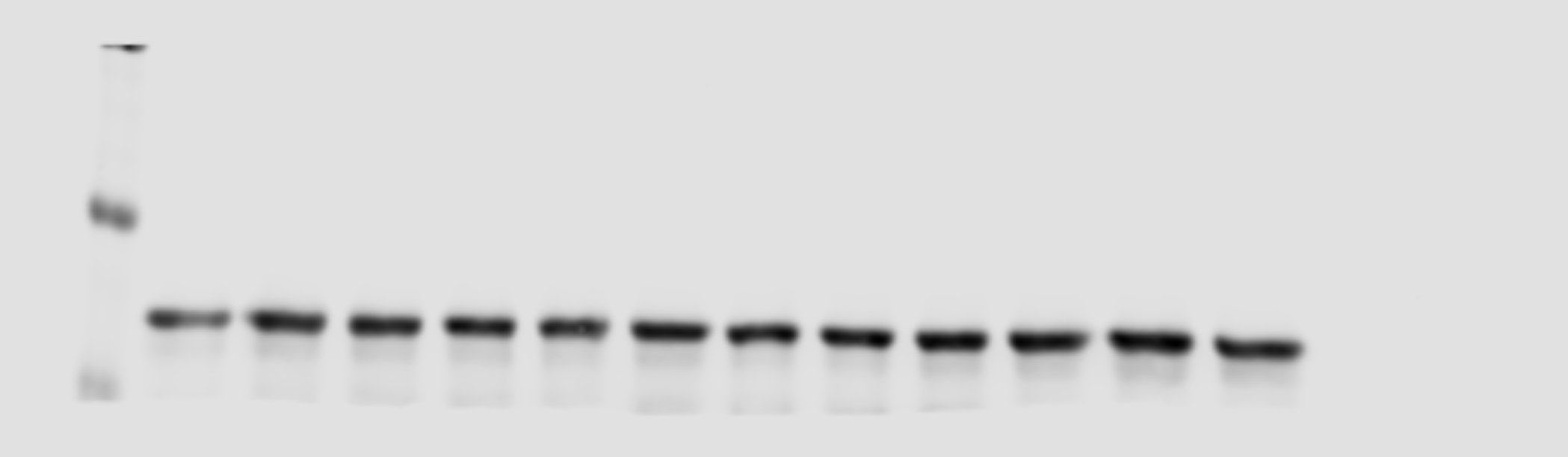

Supplement: Supplementary file 4 — Source Data [file 41467_2022_30060_MOESM4_ESM.zip › source_data/Figure 7/Fig7b_2017-10-10_Metformin_bactin for HMG.tif]

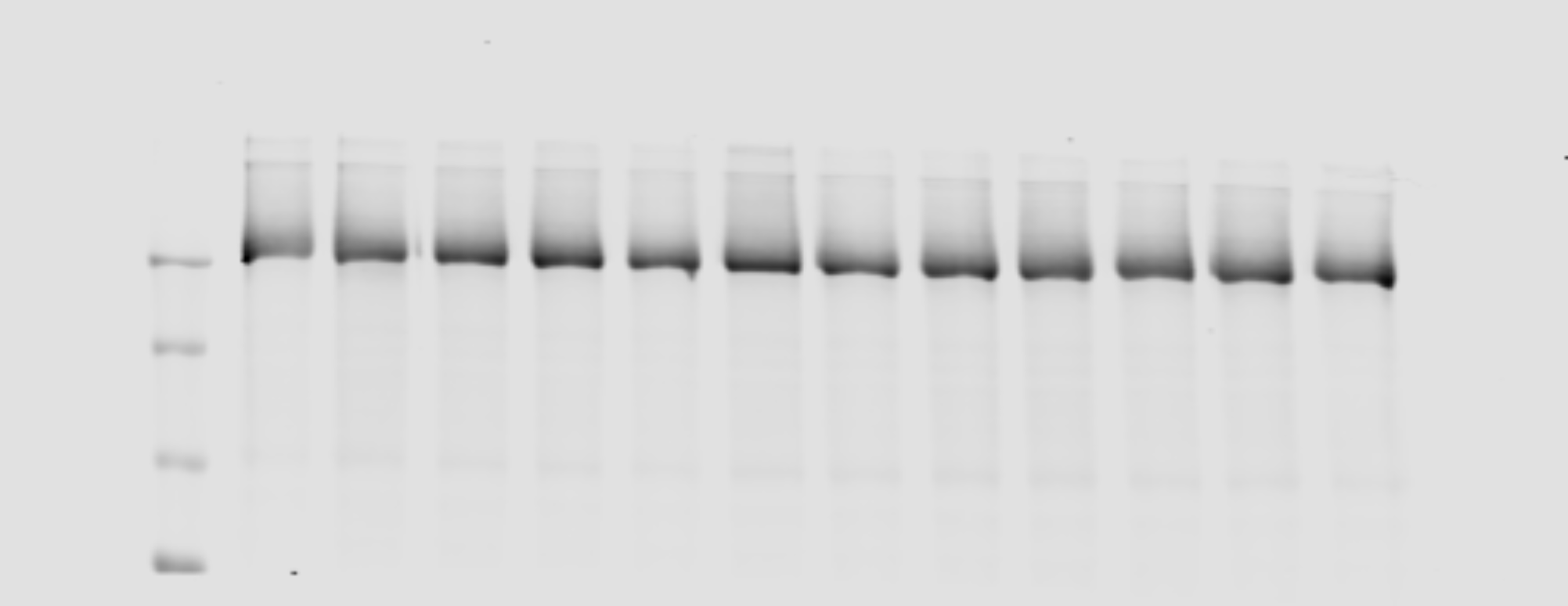

Supplement: Supplementary file 4 — Source Data [file 41467_2022_30060_MOESM4_ESM.zip › source_data/Figure 7/Fig7b_2017-10-11_Metformin_HMGCR_FAS.tif]

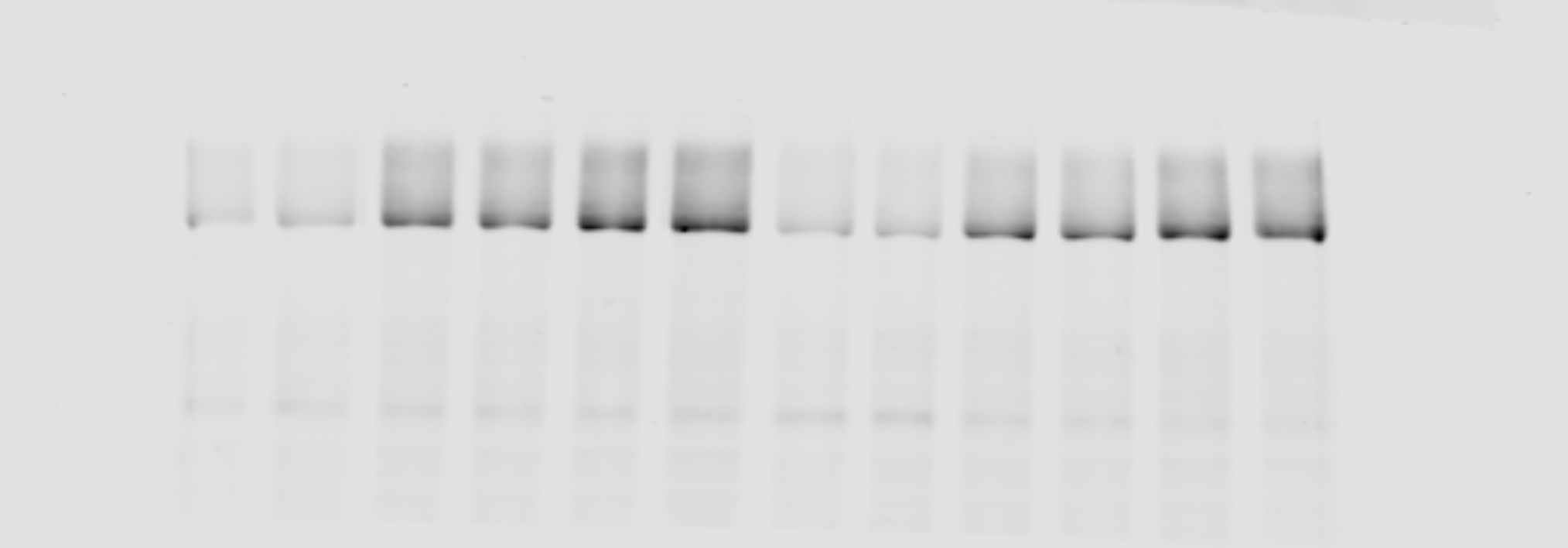

Supplement: Supplementary file 4 — Source Data [file 41467_2022_30060_MOESM4_ESM.zip › source_data/Figure 7/Fig7b_2017-10-10_Metformin_pACC.tif]

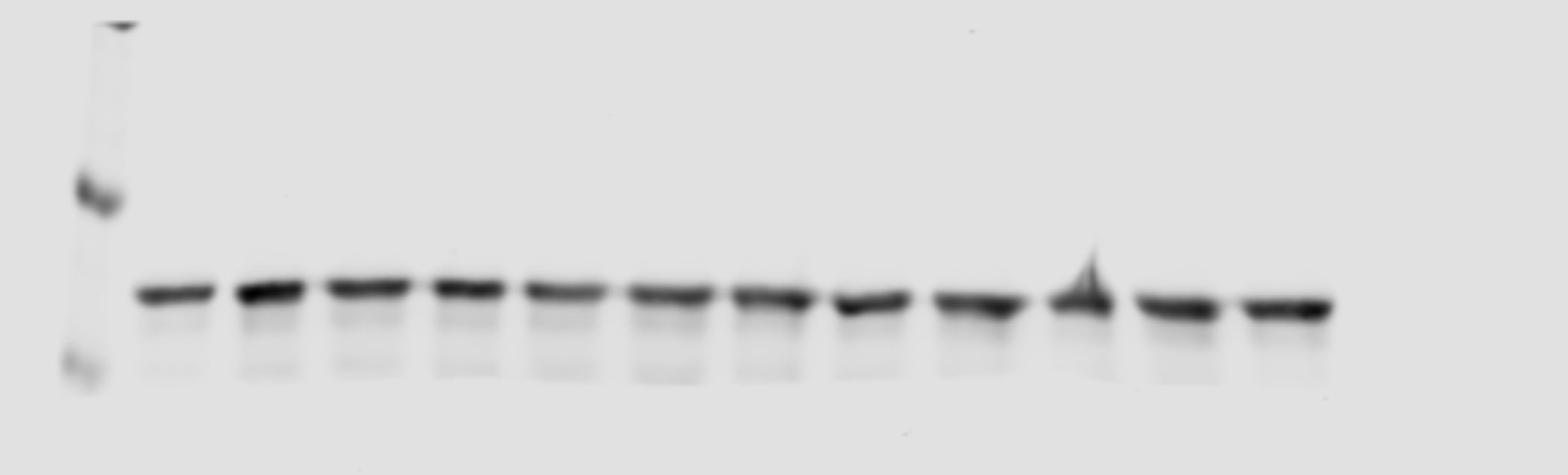

Supplement: Supplementary file 4 — Source Data [file 41467_2022_30060_MOESM4_ESM.zip › source_data/Figure 7/Fig7b_2017-10-10_Metformin_bactin for ACC.tif]

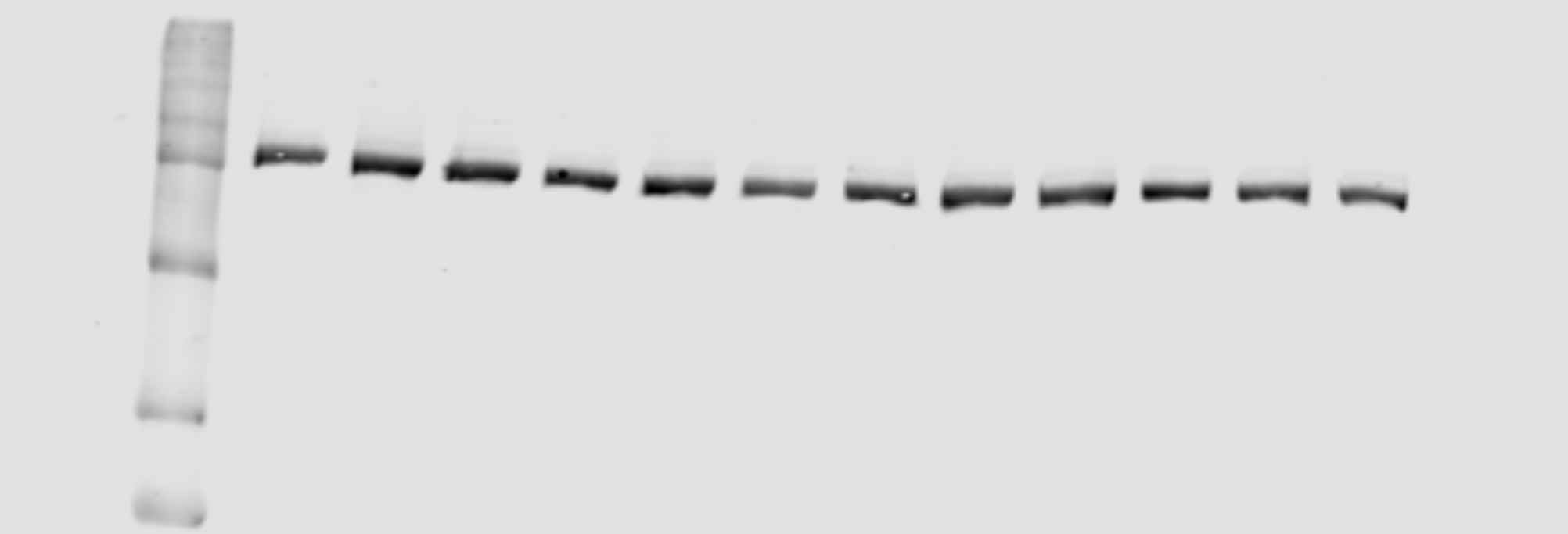

Supplement: Supplementary file 4 — Source Data [file 41467_2022_30060_MOESM4_ESM.zip › source_data/Supplementary Fig/EFig8_2017-10-06_AICAR curve 3_FAS.tif]

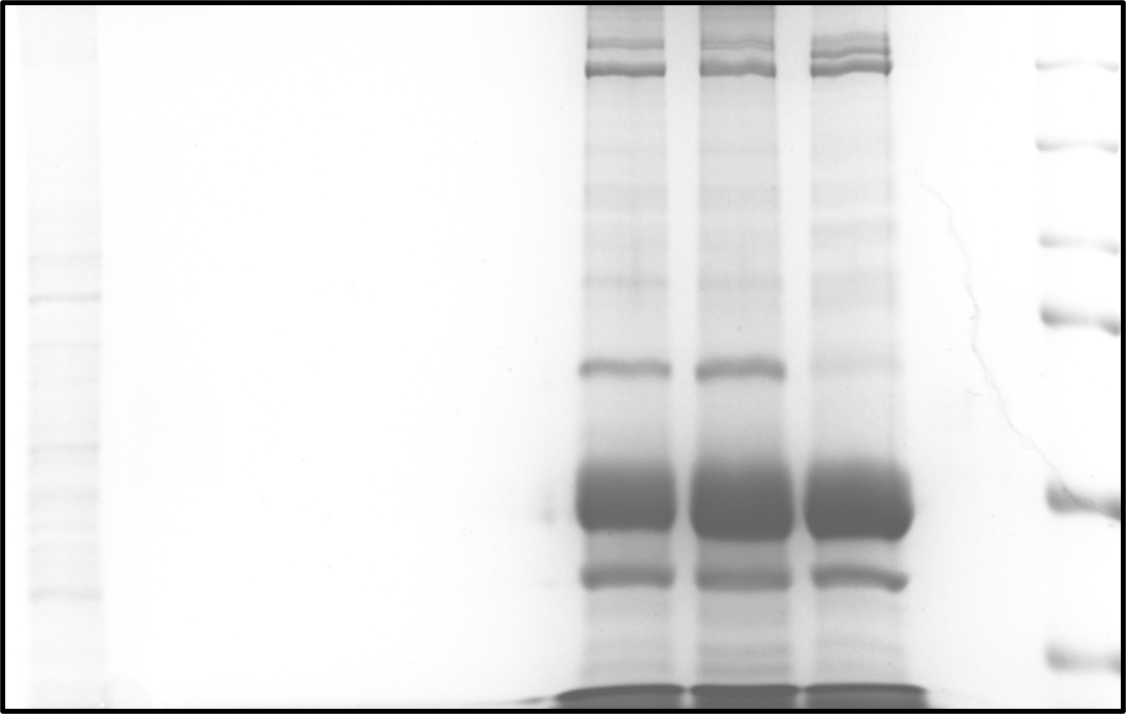

Supplement: Supplementary file 4 — Source Data [file 41467_2022_30060_MOESM4_ESM.zip › source_data/Supplementary Fig/EFig1a.tif]

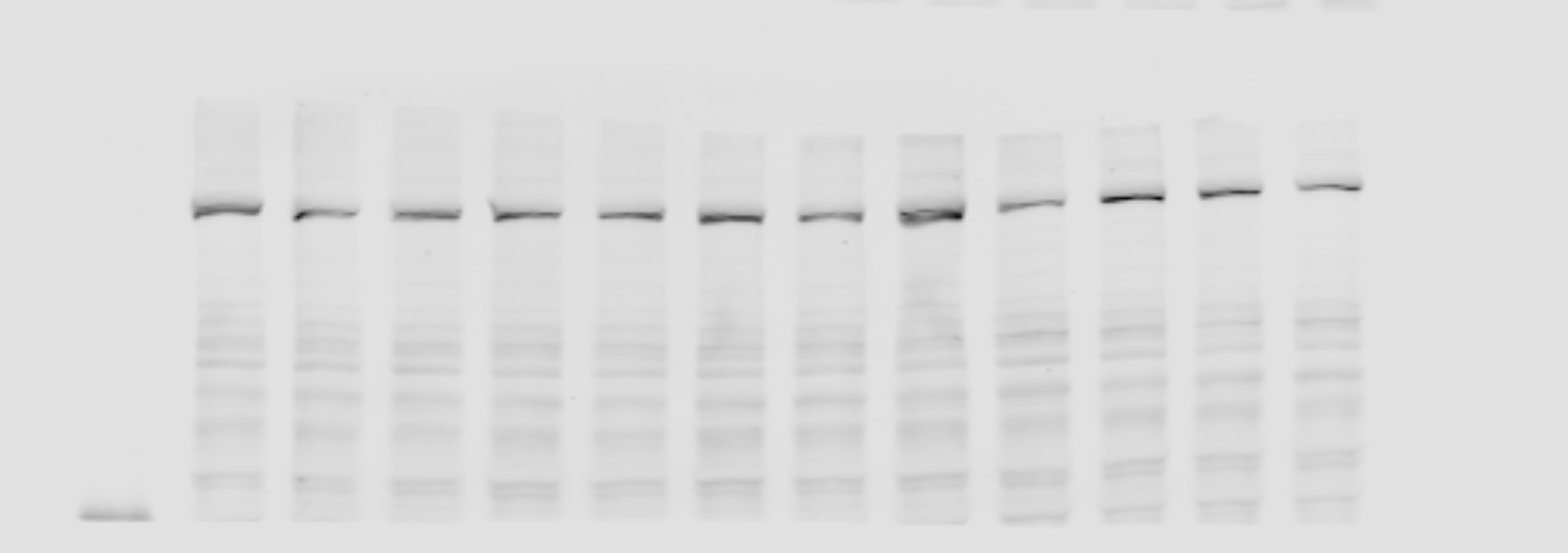

Supplement: Supplementary file 4 — Source Data [file 41467_2022_30060_MOESM4_ESM.zip › source_data/Supplementary Fig/EFig8_2017-10-05_AICAR curve 3_ACC.tif]

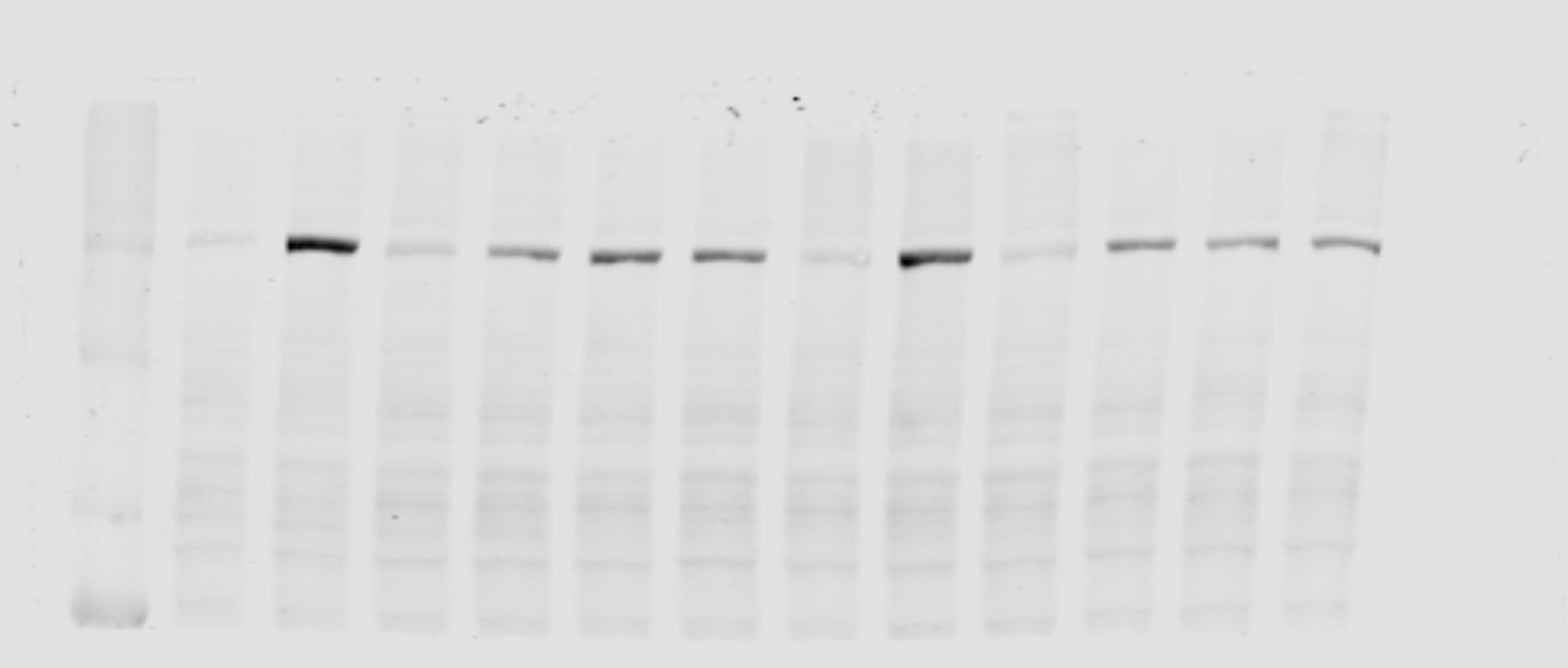

Supplement: Supplementary file 4 — Source Data [file 41467_2022_30060_MOESM4_ESM.zip › source_data/Supplementary Fig/EFig8_2017-10-05_AICAR curve 3_HMG.tif]

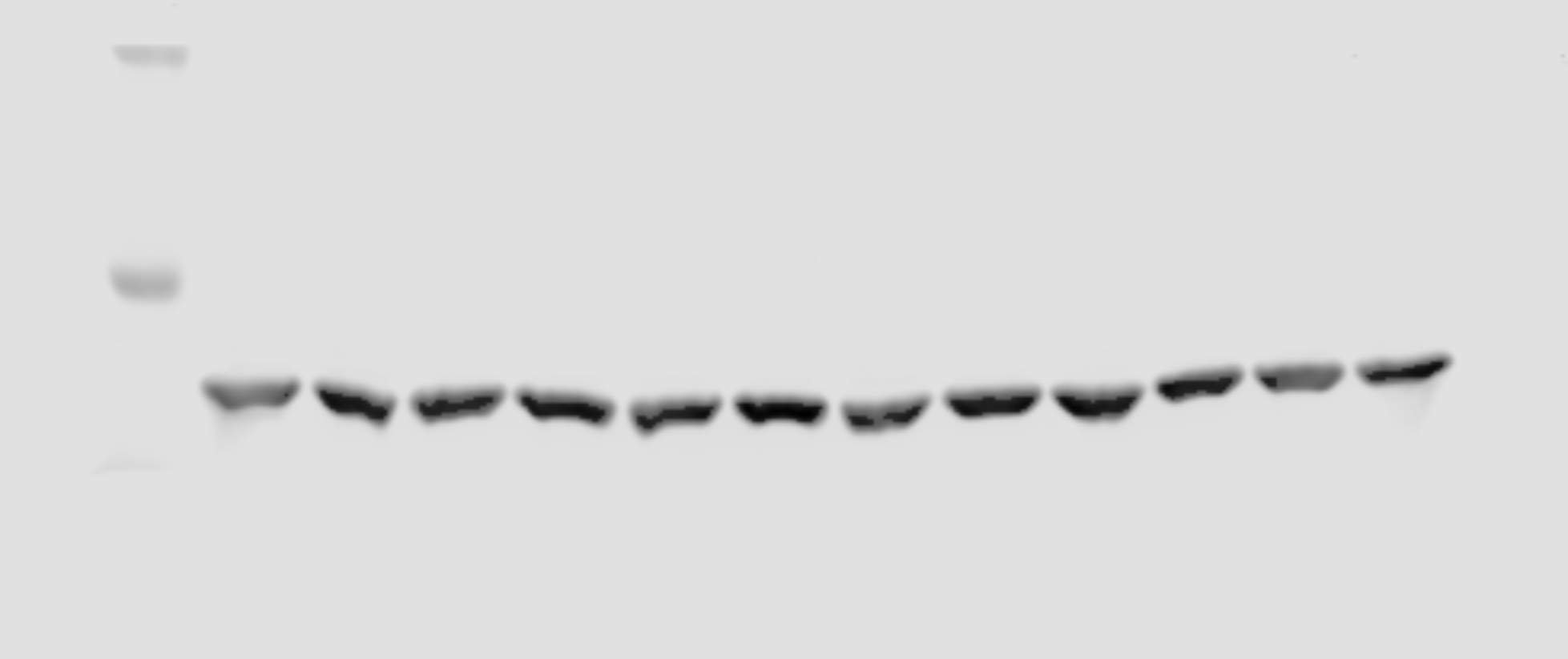

Supplement: Supplementary file 4 — Source Data [file 41467_2022_30060_MOESM4_ESM.zip › source_data/Supplementary Fig/EFig8_2017-10-05_AICAR curve 3_bactin for ACC.tif]

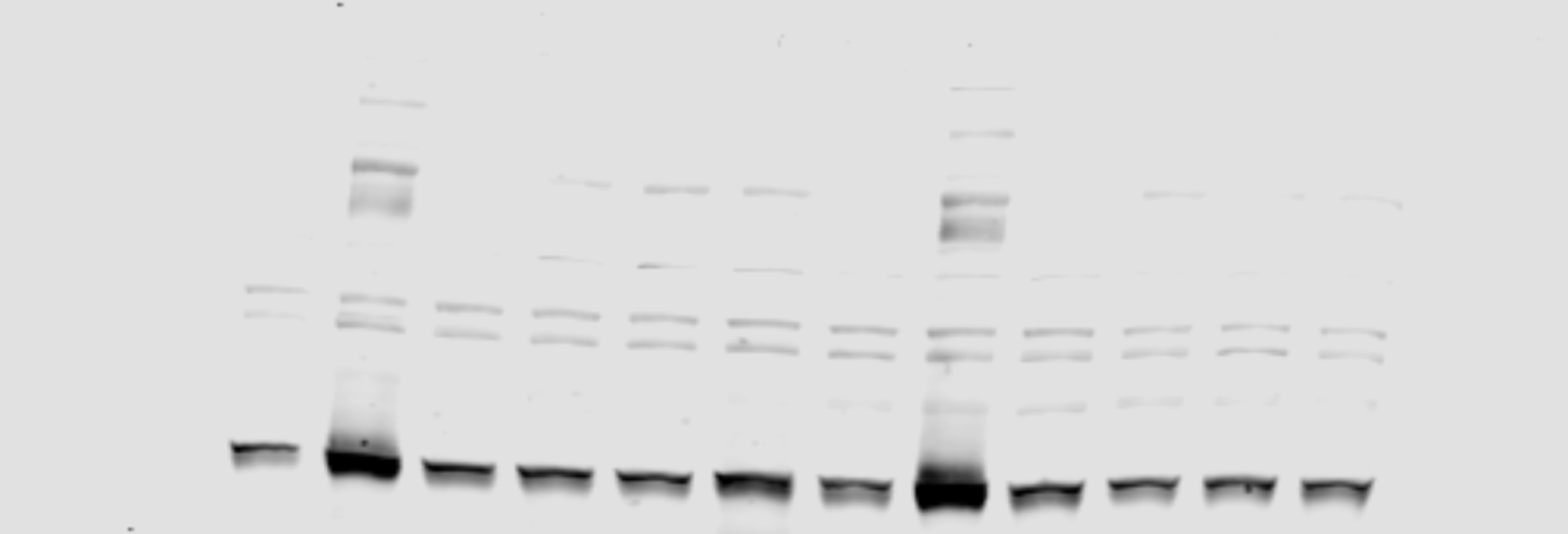

Supplement: Supplementary file 4 — Source Data [file 41467_2022_30060_MOESM4_ESM.zip › source_data/Supplementary Fig/EFig8_2017-10-06_AICAR curve 3_HMGCR.tif]

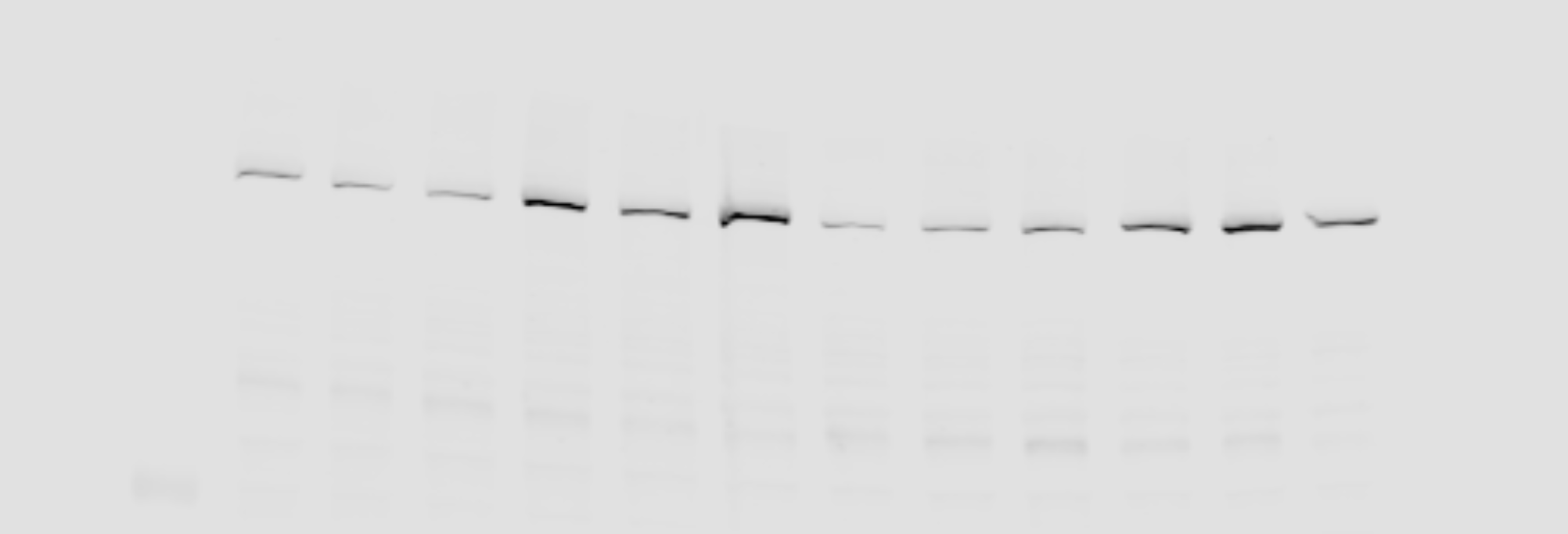

Supplement: Supplementary file 4 — Source Data [file 41467_2022_30060_MOESM4_ESM.zip › source_data/Supplementary Fig/EFig8_2017-10-05_AICAR curve 3_pACC.tif]

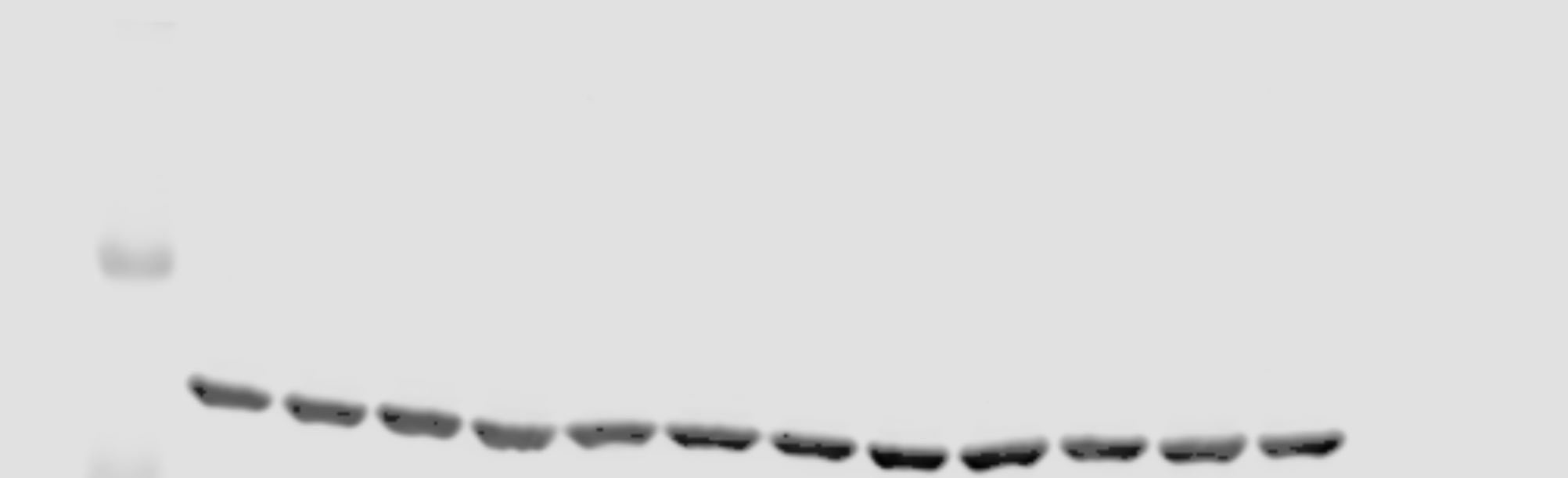

Supplement: Supplementary file 4 — Source Data [file 41467_2022_30060_MOESM4_ESM.zip › source_data/Supplementary Fig/EFig8_2017-10-05_AICAR curve 3_bactin forHMG.tif]

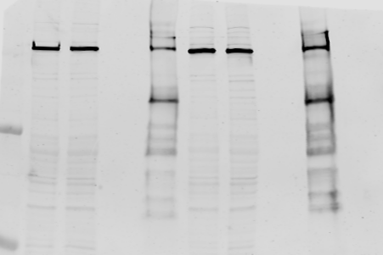

Supplement: Supplementary file 4 — Source Data [file 41467_2022_30060_MOESM4_ESM.zip › source_data/Supplementary Fig/EF6_FAS for HMG blot.tif]

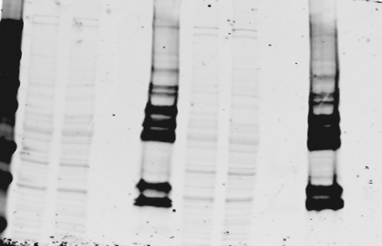

Supplement: Supplementary file 4 — Source Data [file 41467_2022_30060_MOESM4_ESM.zip › source_data/Supplementary Fig/EF6_HMG blot.tif]

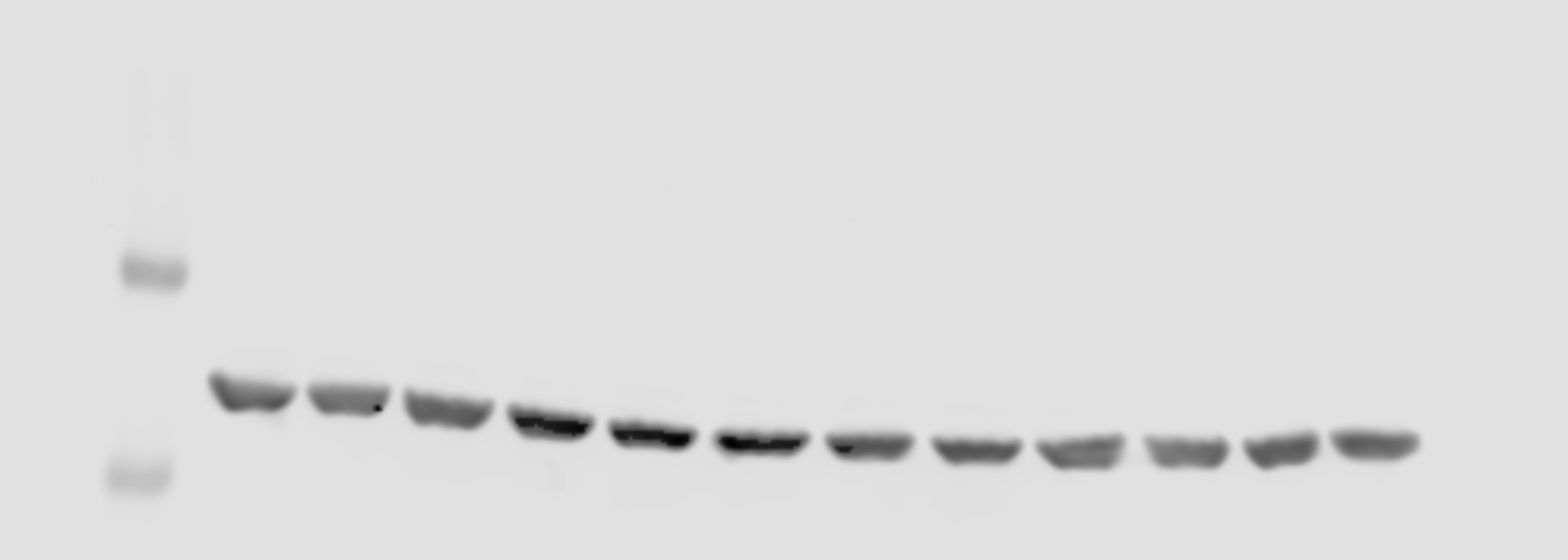

Supplement: Supplementary file 4 — Source Data [file 41467_2022_30060_MOESM4_ESM.zip › source_data/Supplementary Fig/EFig8_2017-10-05_AICAR curve 3_bactin for pACC.tif]

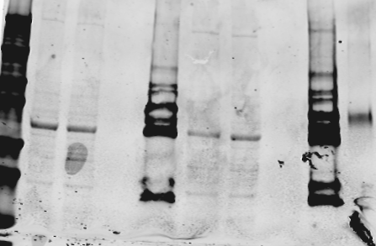

Supplement: Supplementary file 4 — Source Data [file 41467_2022_30060_MOESM4_ESM.zip › source_data/Supplementary Fig/EF6_HMG competition blot.tif]

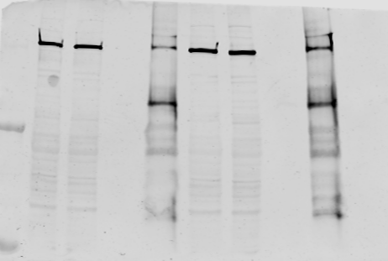

Supplement: Supplementary file 4 — Source Data [file 41467_2022_30060_MOESM4_ESM.zip › source_data/Supplementary Fig/EF6_FAS for HMG competition blot.tif]

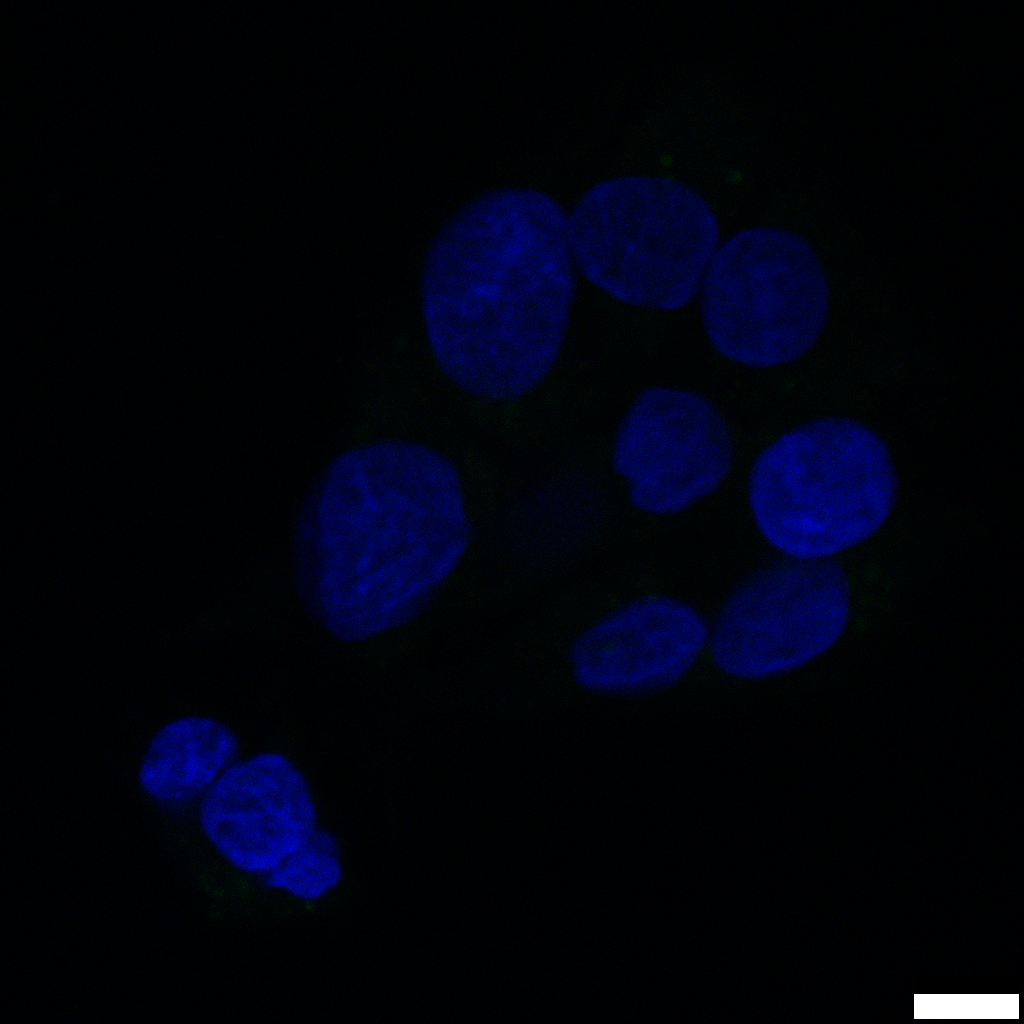

Supplement: Supplementary file 4 — Source Data [file 41467_2022_30060_MOESM4_ESM.zip › source_data/Figure 6/No probes.tif]

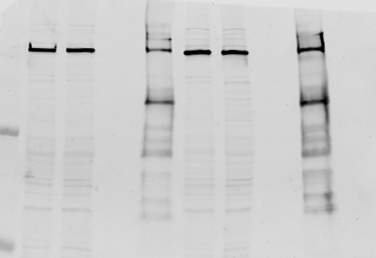

Supplement: Supplementary file 4 — Source Data [file 41467_2022_30060_MOESM4_ESM.zip › source_data/Figure 6/Fig6e_FAS.tif]

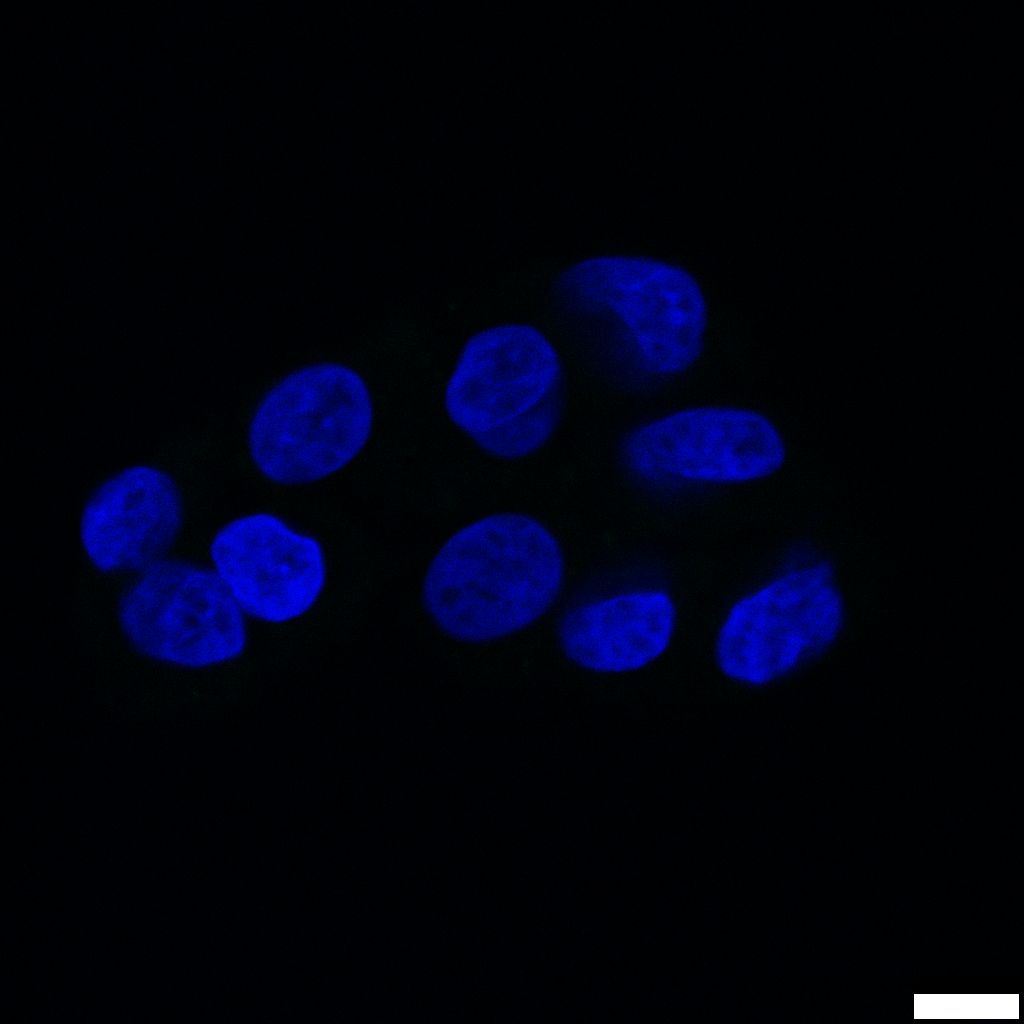

Supplement: Supplementary file 4 — Source Data [file 41467_2022_30060_MOESM4_ESM.zip › source_data/Figure 6/HMGCR only.tif]

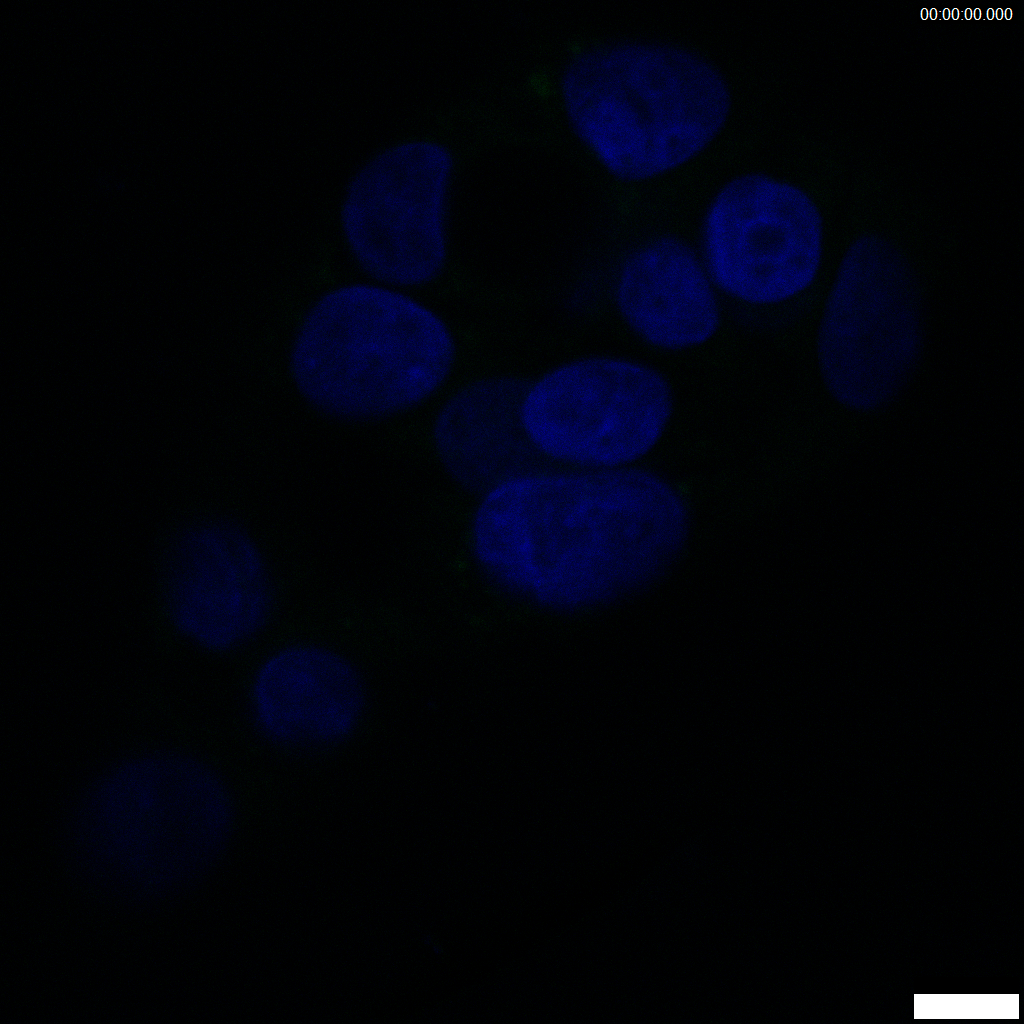

Supplement: Supplementary file 4 — Source Data [file 41467_2022_30060_MOESM4_ESM.zip › source_data/Figure 6/FAS only.tif]

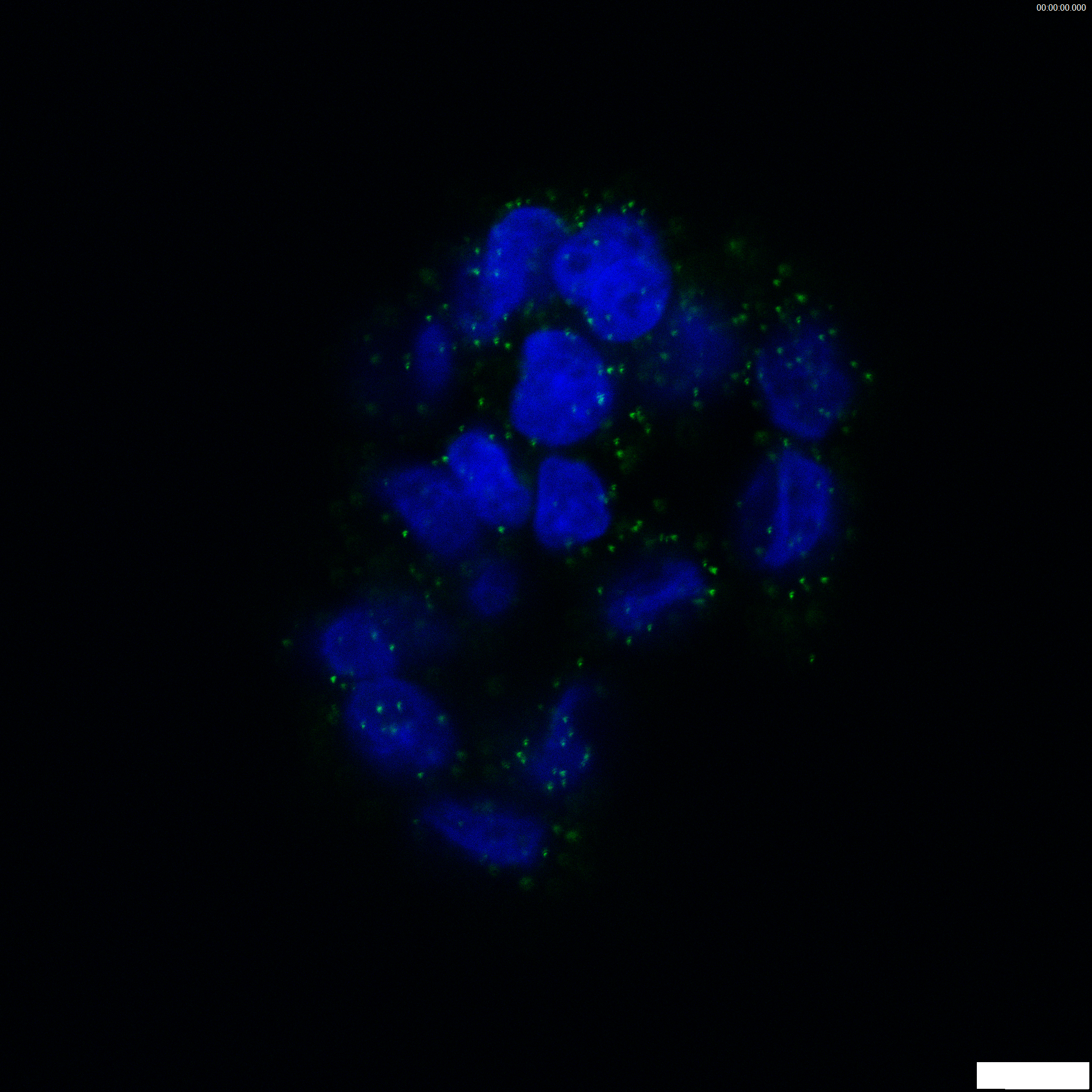

Supplement: Supplementary file 4 — Source Data [file 41467_2022_30060_MOESM4_ESM.zip › source_data/Figure 6/FAS+HMGCR.tif]

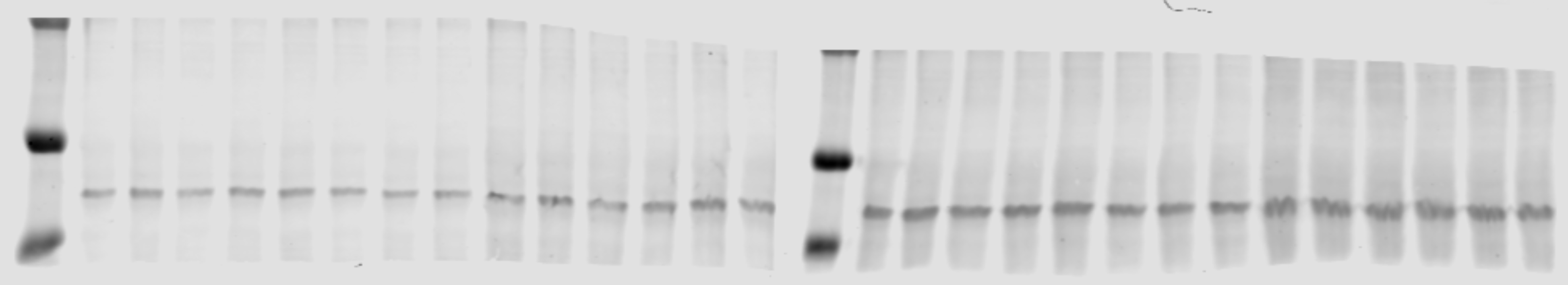

Supplement: Supplementary file 4 — Source Data [file 41467_2022_30060_MOESM4_ESM.zip › source_data/Figure 1/Fig1d_betaactin_2018-11-16_StatinMouse5_blot3_bactin.tif]

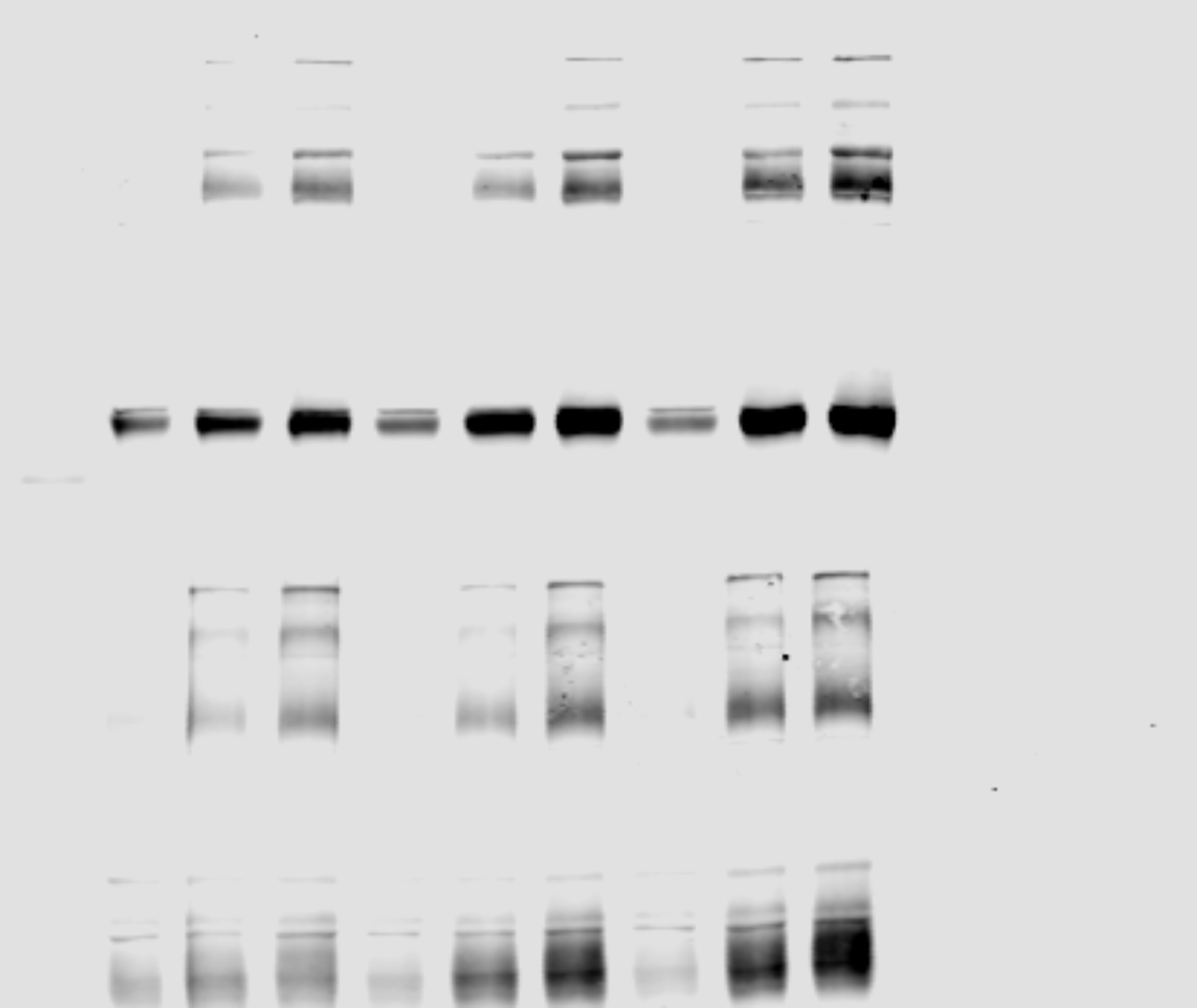

Supplement: Supplementary file 4 — Source Data [file 41467_2022_30060_MOESM4_ESM.zip › source_data/Figure 1/Fig1b_HMGCR_2018-12-13_StatinTimeCourseSet2_HMGCR.tif]

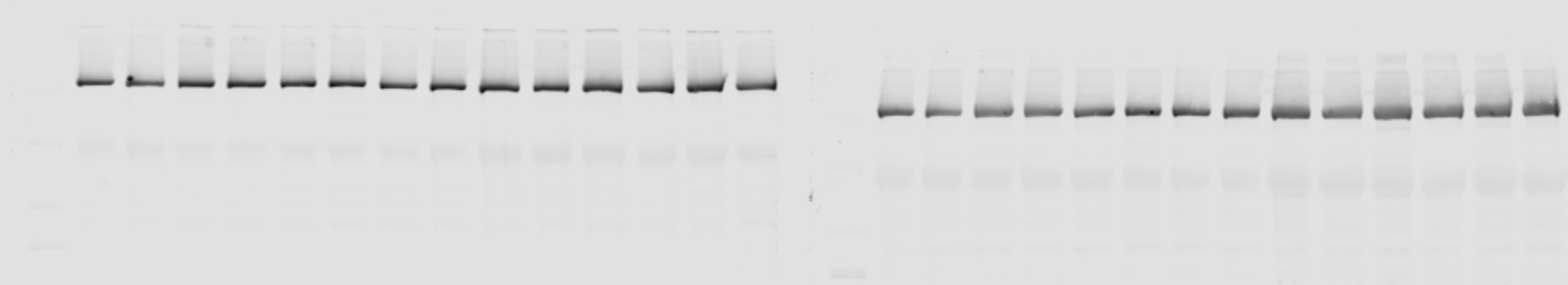

Supplement: Supplementary file 4 — Source Data [file 41467_2022_30060_MOESM4_ESM.zip › source_data/Figure 1/Fig1d_FAS_2018-11-16_StatinMouse5_blot3_FAS.tif]

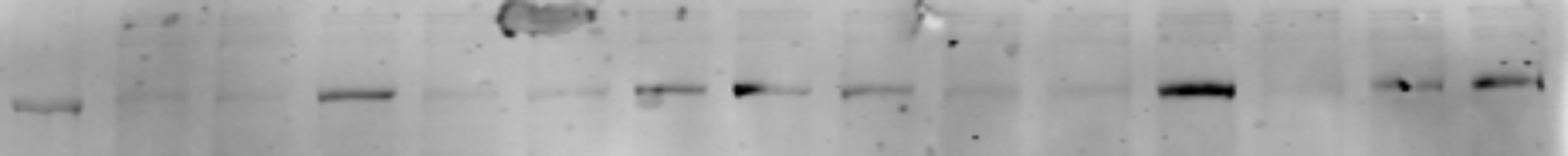

Supplement: Supplementary file 4 — Source Data [file 41467_2022_30060_MOESM4_ESM.zip › source_data/Figure 1/Fig1d_HMGylation_2018-11-08_StatinMouse5_blot3_HMG_frozen.tif]

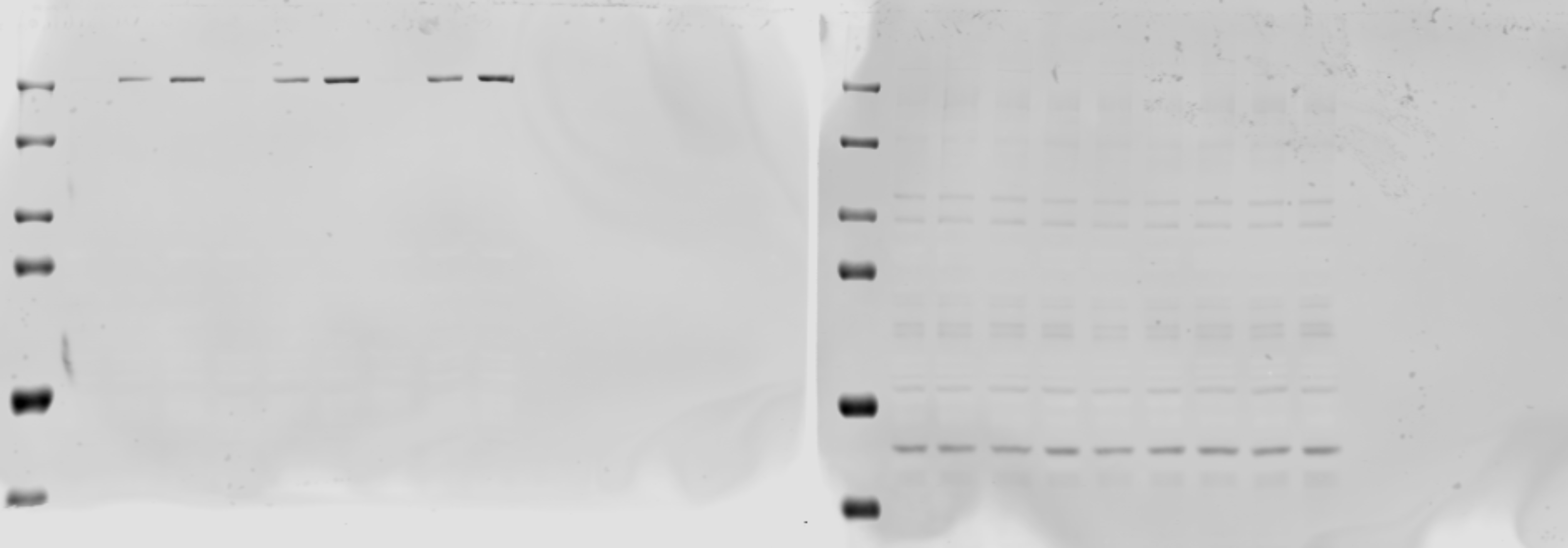

Supplement: Supplementary file 4 — Source Data [file 41467_2022_30060_MOESM4_ESM.zip › source_data/Figure 1/Fig1b_HMGylation_2018-11-28_StatinTimeCourseSet2_HMGComp.tif]

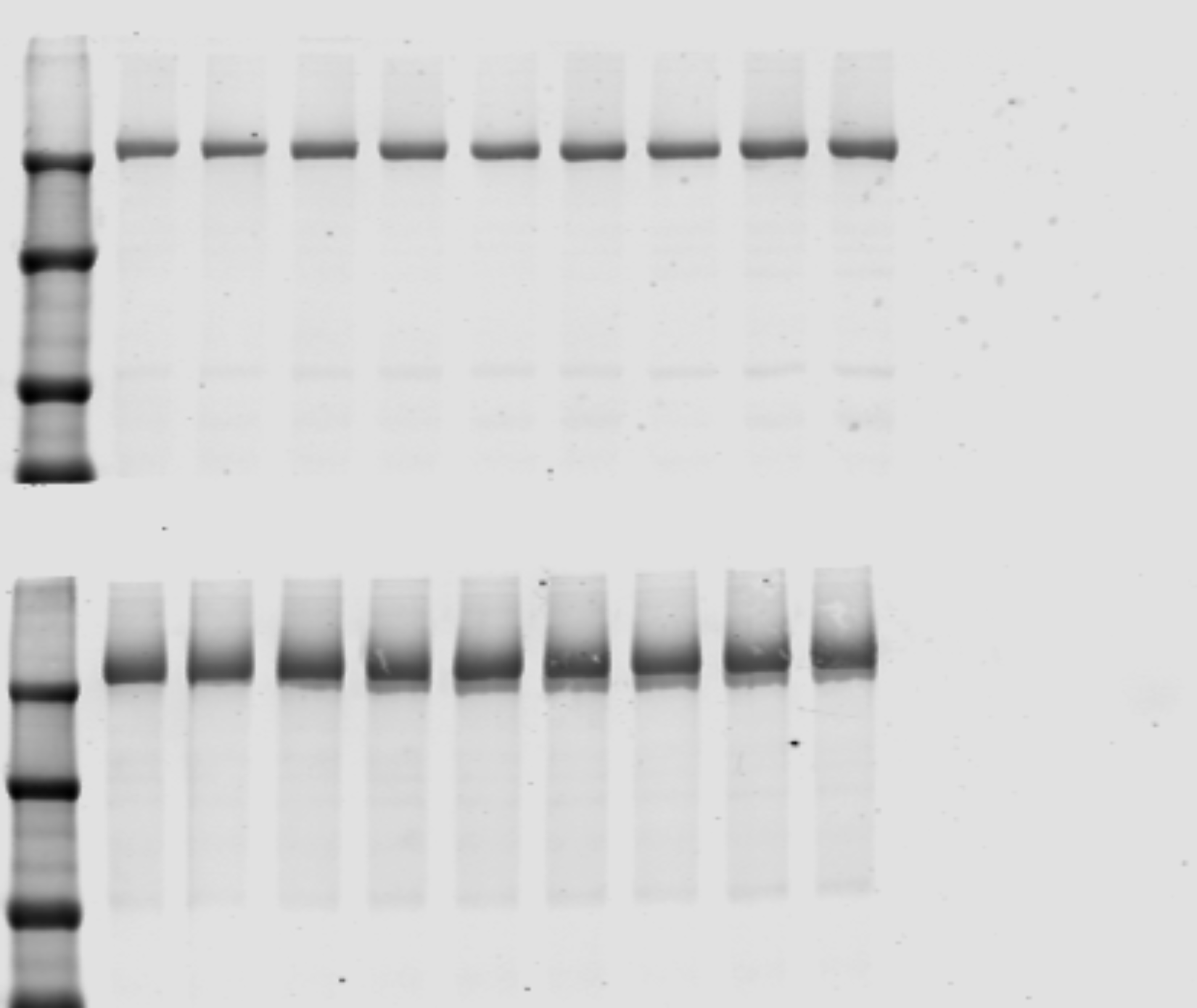

Supplement: Supplementary file 4 — Source Data [file 41467_2022_30060_MOESM4_ESM.zip › source_data/Figure 1/Fig1b_FAS_2018-12-13_StatinTimeCourseSet2_FAS.tif]

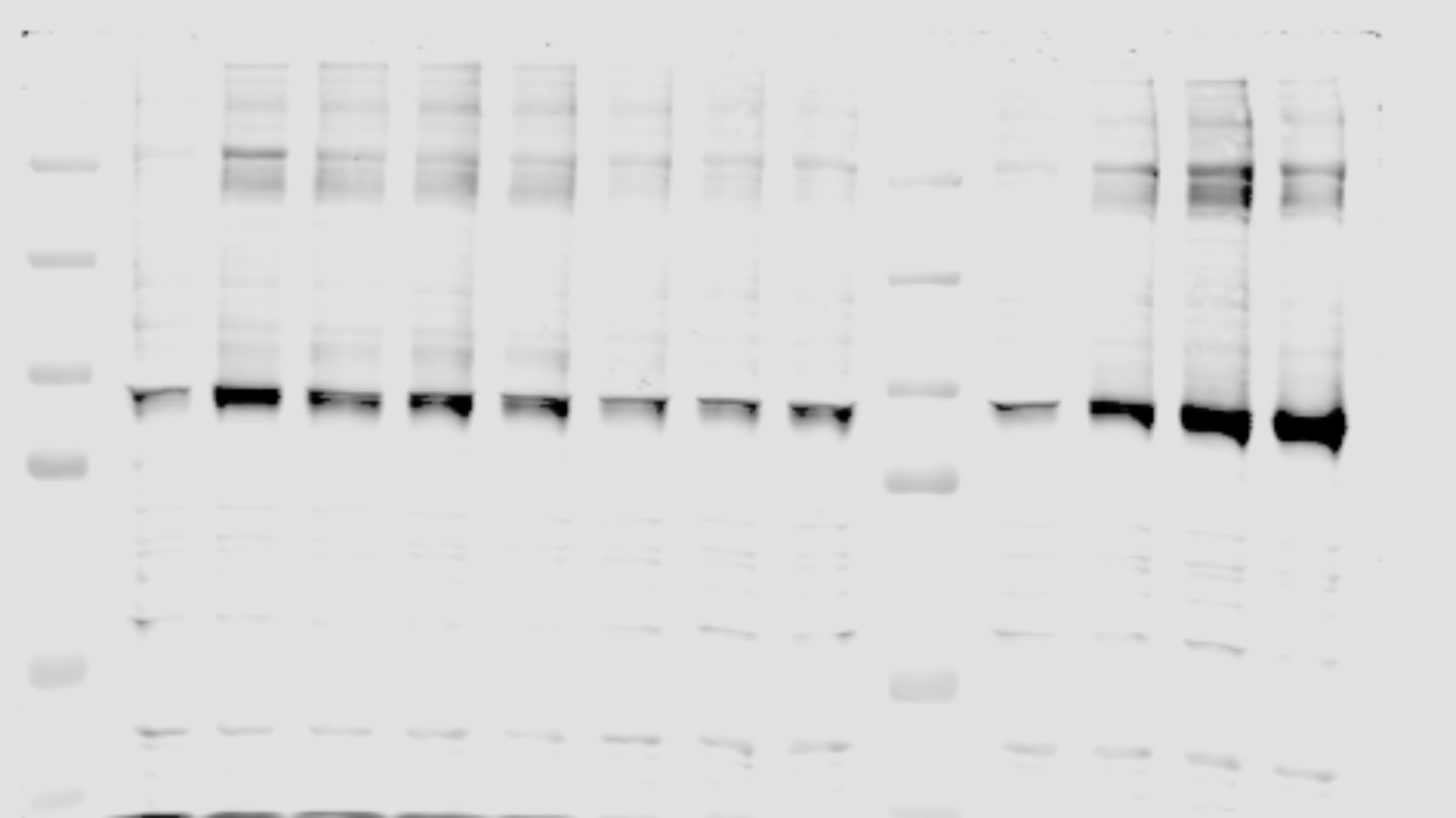

Supplement: Supplementary file 4 — Source Data [file 41467_2022_30060_MOESM4_ESM.zip › source_data/Figure 4/Fig4d_2017-01-23_Statin withdrawl adn time course_HMGCR.tif]

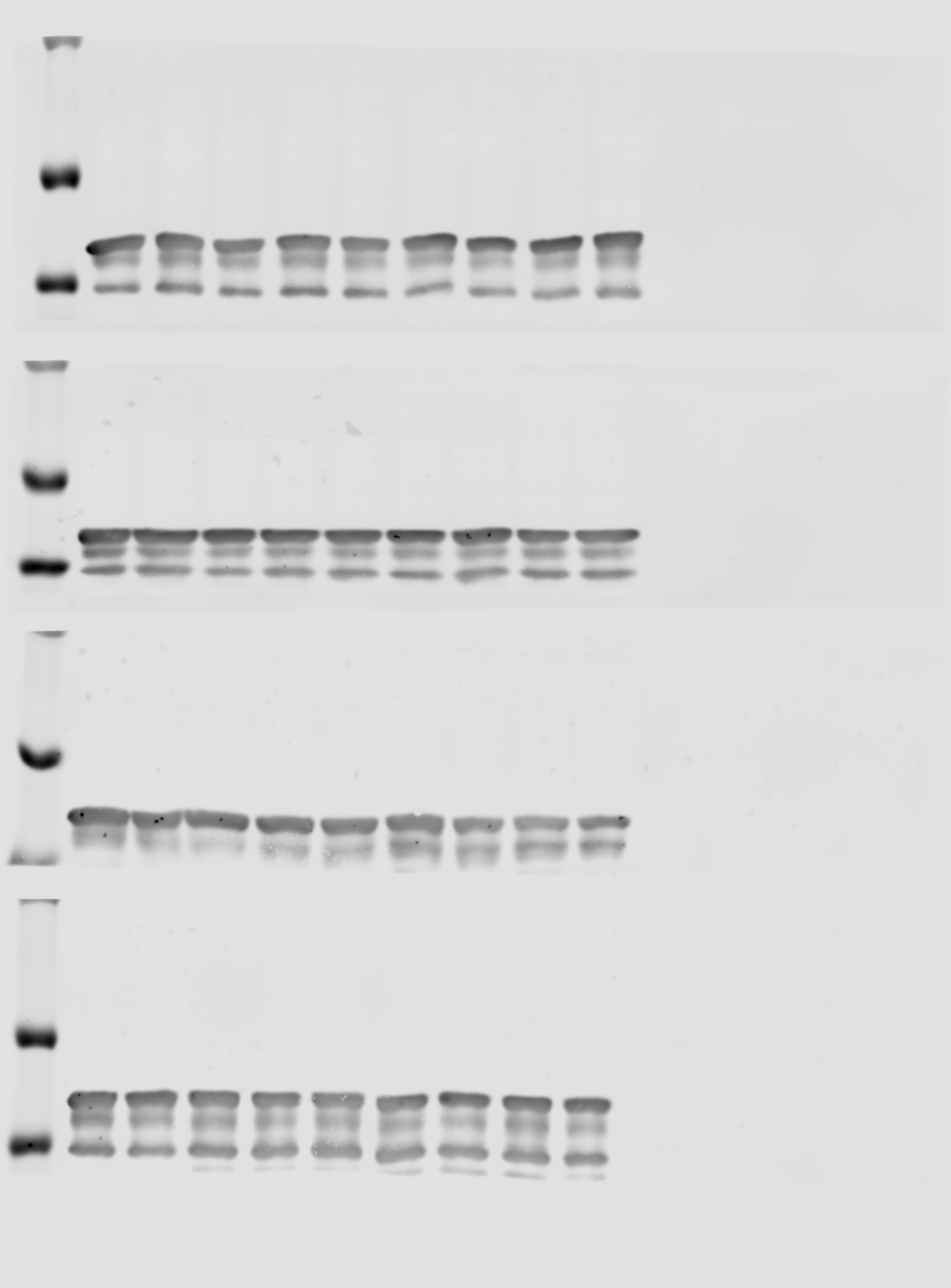

Supplement: Supplementary file 4 — Source Data [file 41467_2022_30060_MOESM4_ESM.zip › source_data/Figure 4/Fig4f_2020-09-18_HA4-bactin.tif]

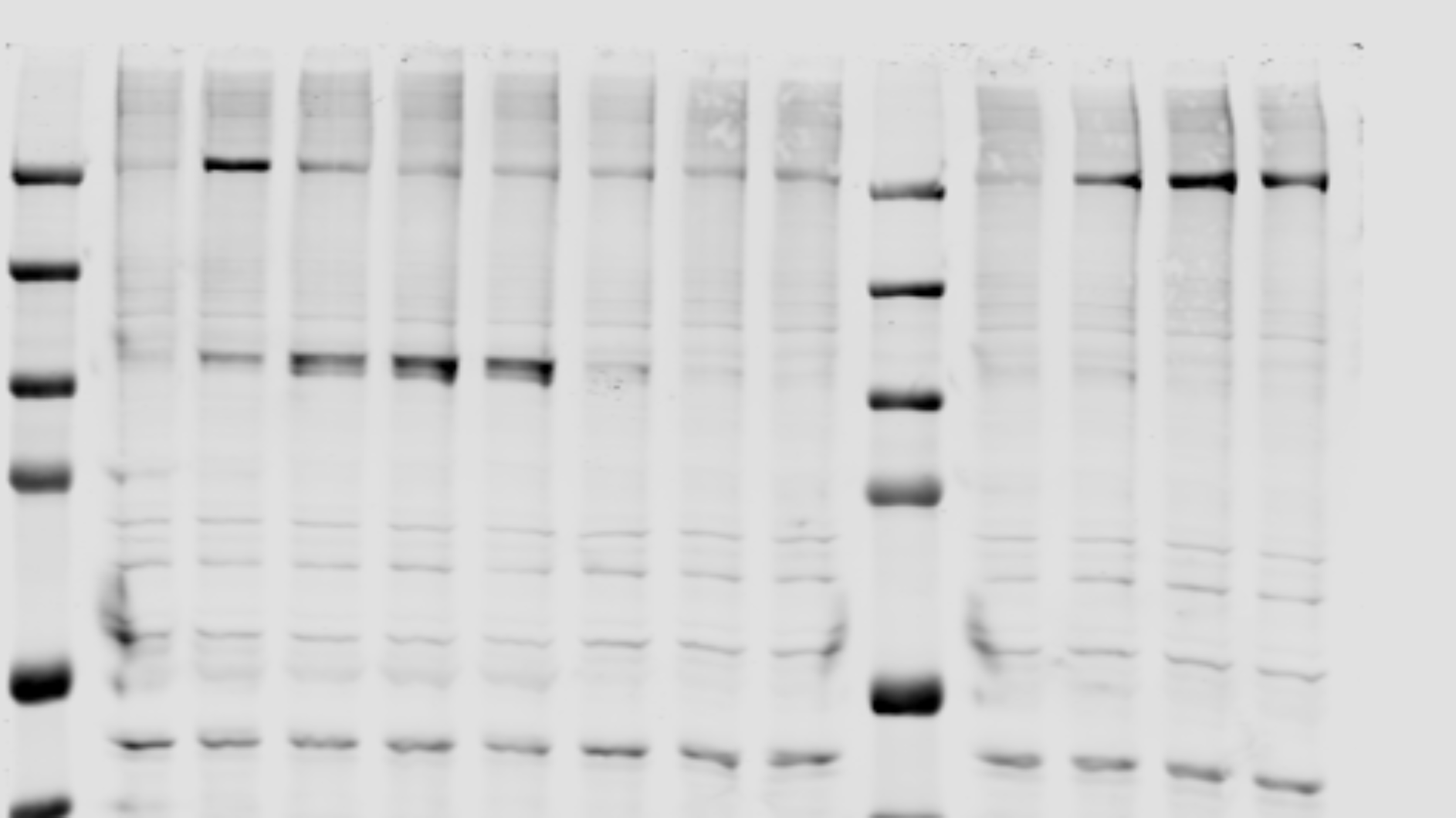

Supplement: Supplementary file 4 — Source Data [file 41467_2022_30060_MOESM4_ESM.zip › source_data/Figure 4/Fig4d_2017-01-21_Statin withdrawal and time course_HMG.tif]

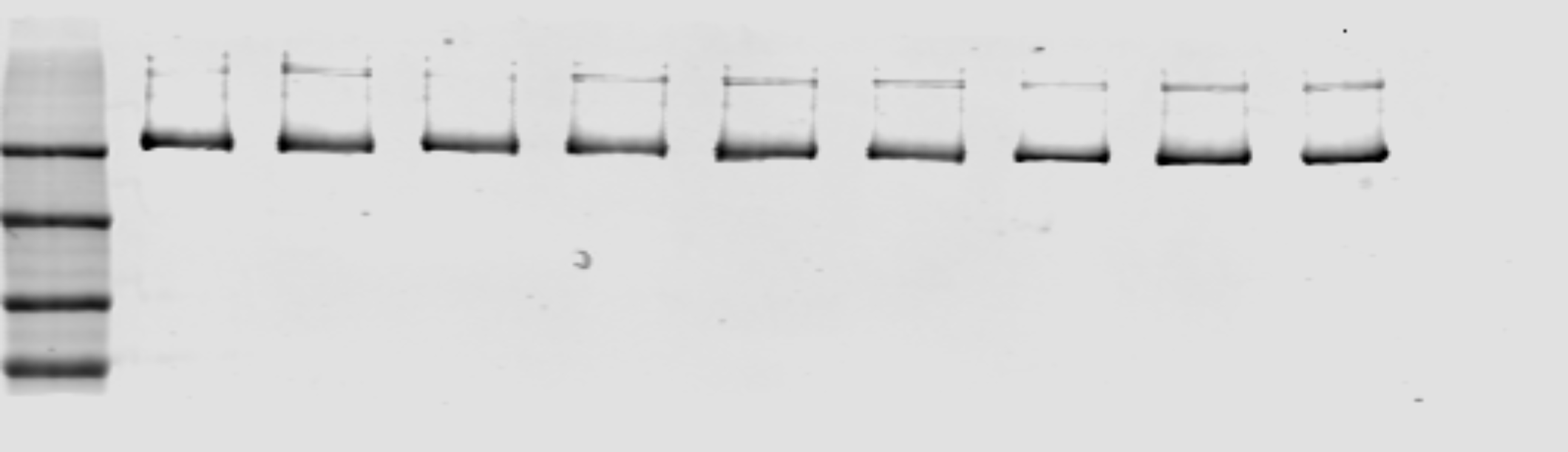

Supplement: Supplementary file 4 — Source Data [file 41467_2022_30060_MOESM4_ESM.zip › source_data/Figure 4/Fig4a_FAS_2016-05-02_HMG Mal_FAS.tif]

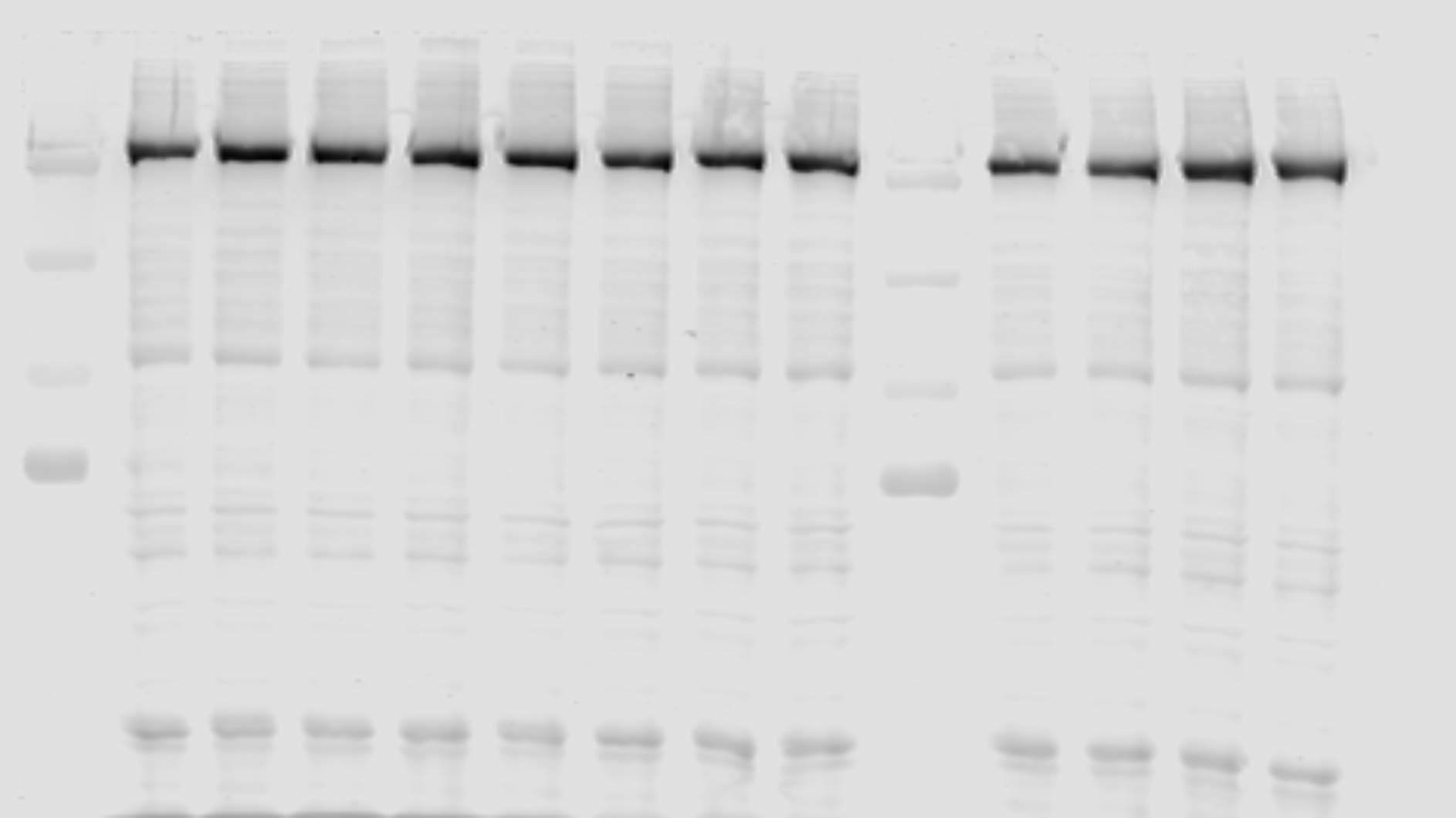

Supplement: Supplementary file 4 — Source Data [file 41467_2022_30060_MOESM4_ESM.zip › source_data/Figure 4/Fig4d_2017-01-23_Statin withdrawl adn time course_FAS.tif]

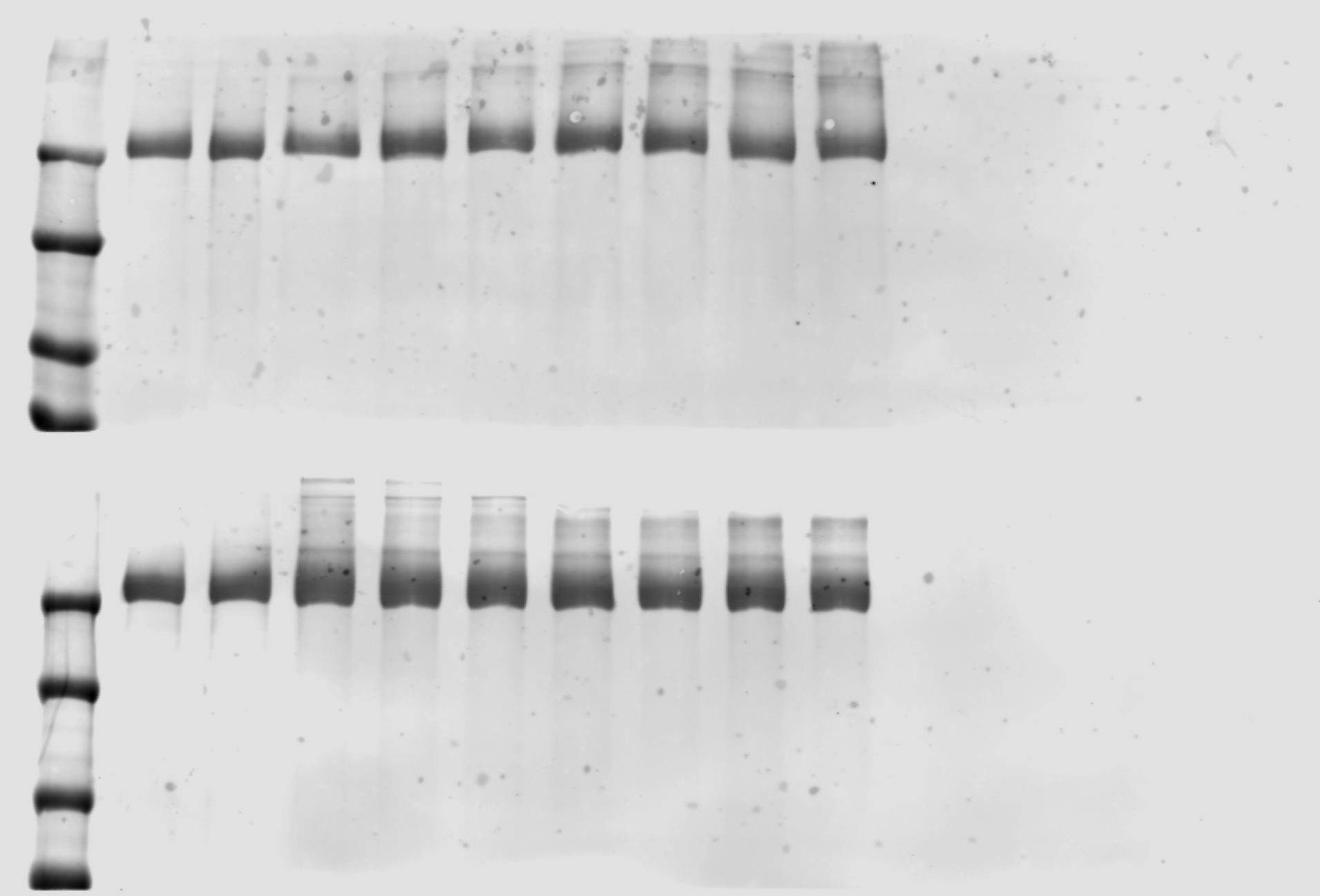

Supplement: Supplementary file 4 — Source Data [file 41467_2022_30060_MOESM4_ESM.zip › source_data/Figure 4/Fig4f_2020-09-18_HA4-FAS_ph96.tif]

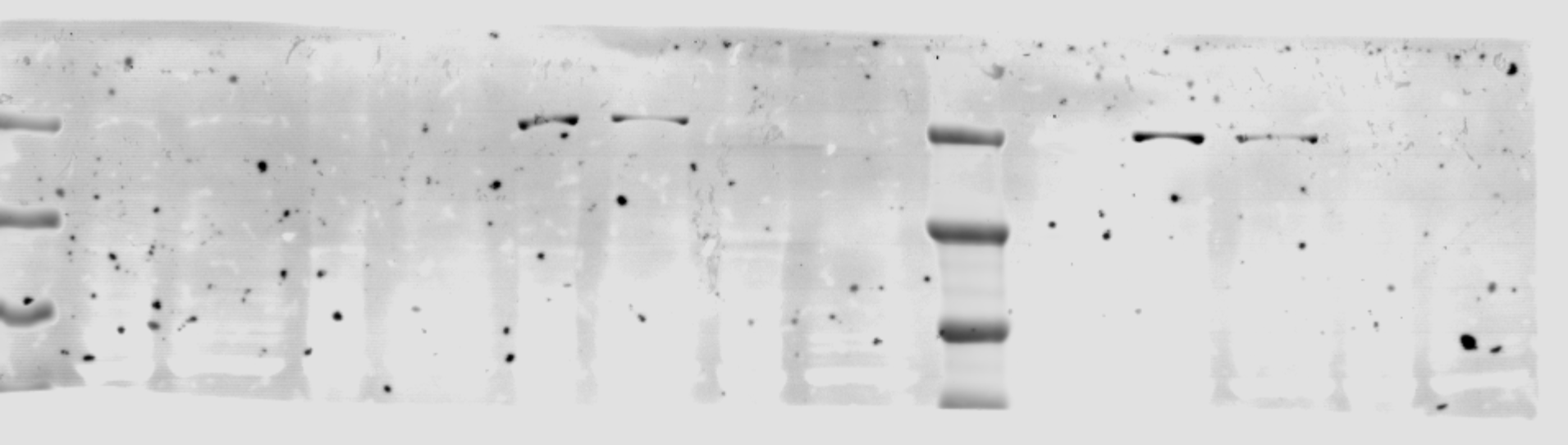

Supplement: Supplementary file 4 — Source Data [file 41467_2022_30060_MOESM4_ESM.zip › source_data/Figure 4/Fig4e_2020-09-18_HeatRed3_HMG.tif]

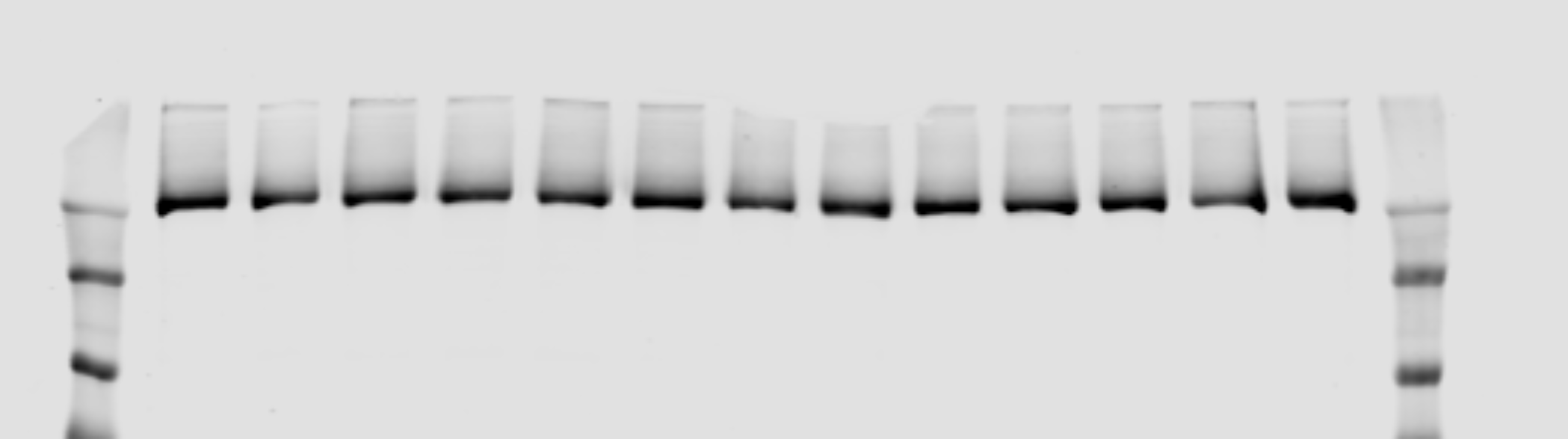

Supplement: Supplementary file 4 — Source Data [file 41467_2022_30060_MOESM4_ESM.zip › source_data/Figure 4/Fig4c_2017-04-26_Statin short course 3_FAS.tif]

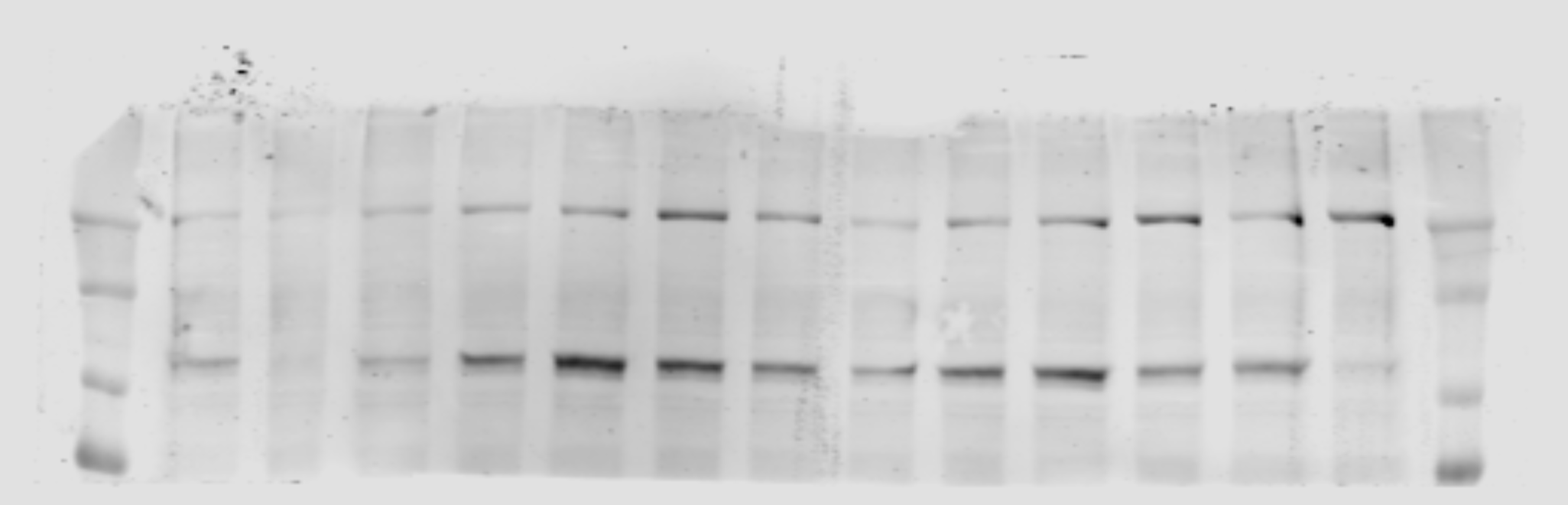

Supplement: Supplementary file 4 — Source Data [file 41467_2022_30060_MOESM4_ESM.zip › source_data/Figure 4/Fig4c_2017-04-25_Statin short course 3_HMG_rescan.tif]

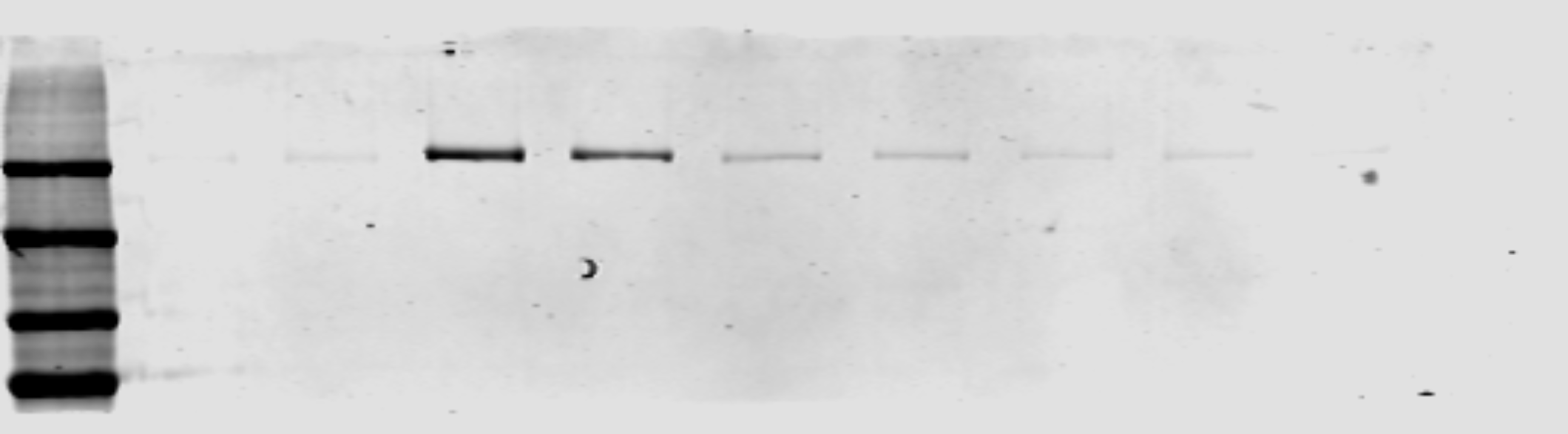

Supplement: Supplementary file 4 — Source Data [file 41467_2022_30060_MOESM4_ESM.zip › source_data/Figure 4/Fig4a_HMG_2016-04-29_HMG Mal_HMG.tif]

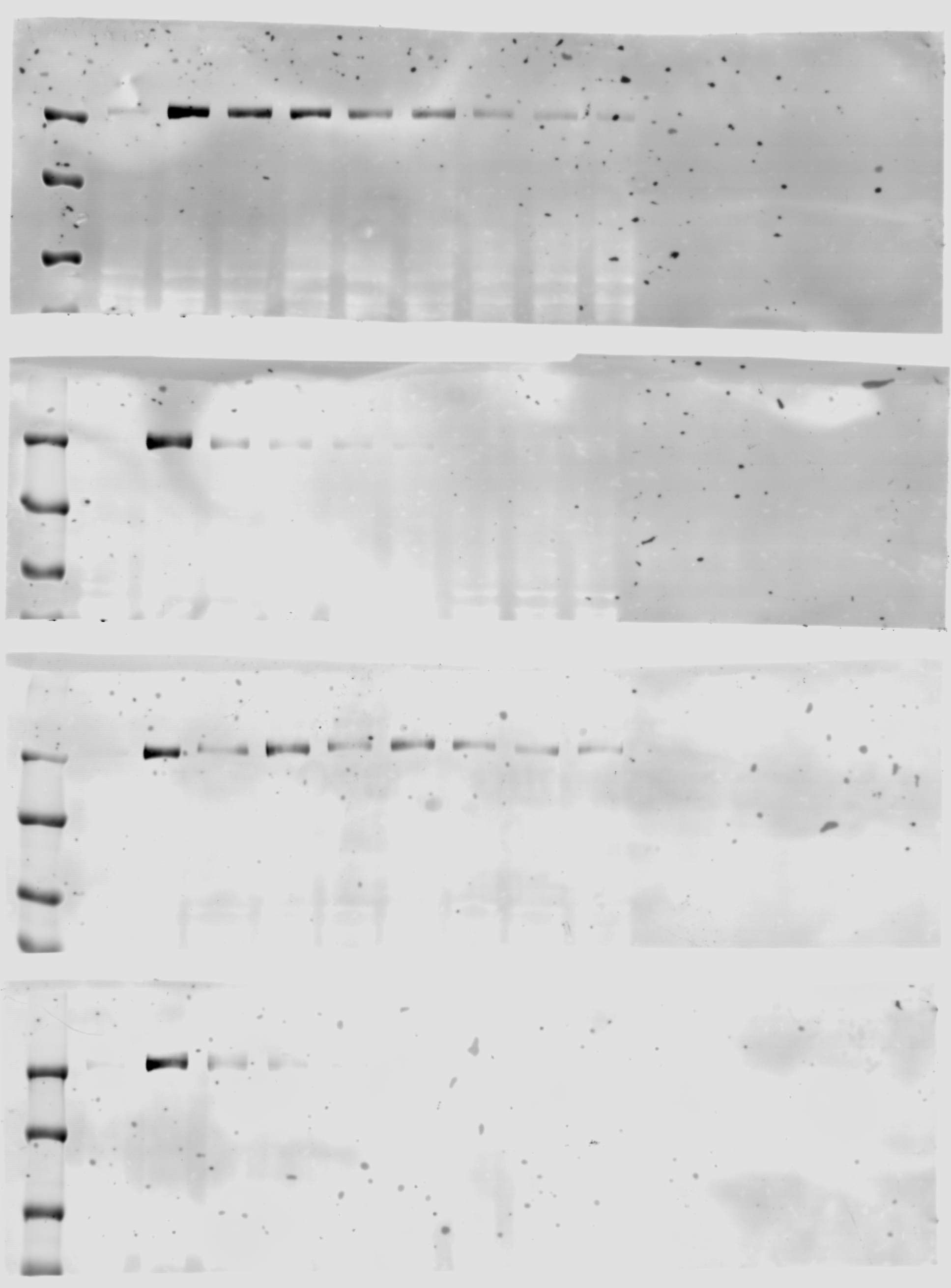

Supplement: Supplementary file 4 — Source Data [file 41467_2022_30060_MOESM4_ESM.zip › source_data/Figure 4/Fig4f_2020-09-18_HA4_HMG.tif]

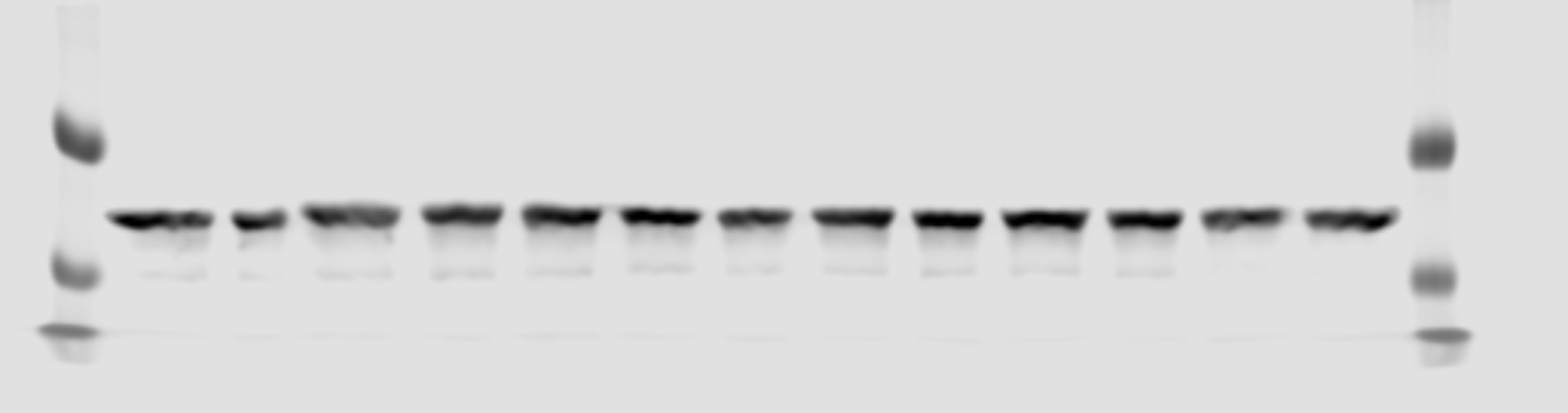

Supplement: Supplementary file 4 — Source Data [file 41467_2022_30060_MOESM4_ESM.zip › source_data/Figure 4/Fig4c_2017-04-25_Statin short course 3_b actin.tif]

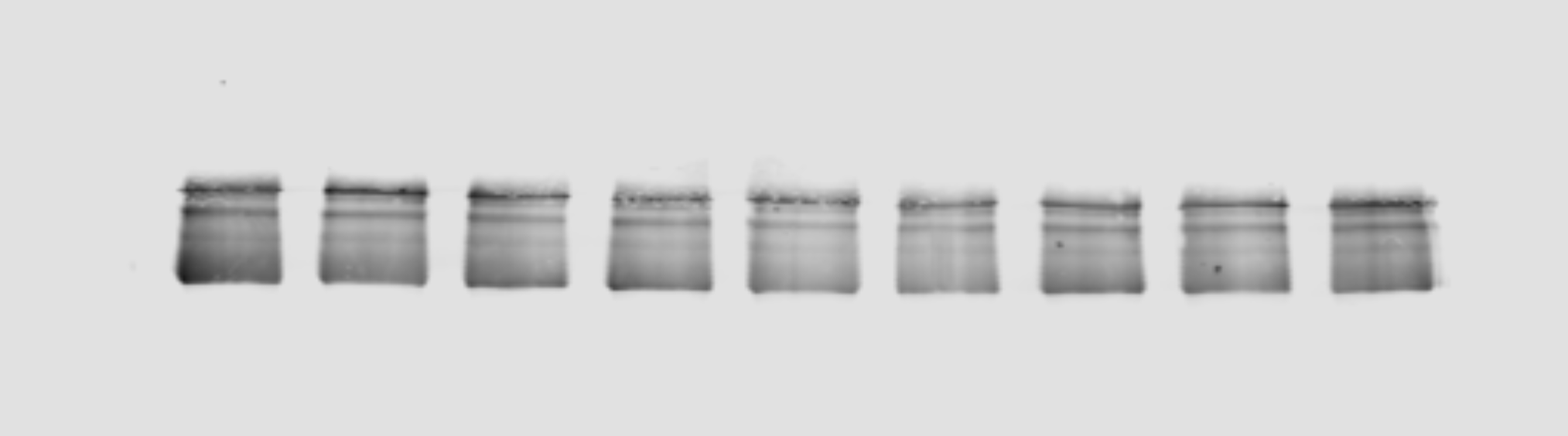

Supplement: Supplementary file 4 — Source Data [file 41467_2022_30060_MOESM4_ESM.zip › source_data/Figure 4/Fig4b_FAS_2016-08-06_HMG pre load_Mal Comp_FAS signal.tif]

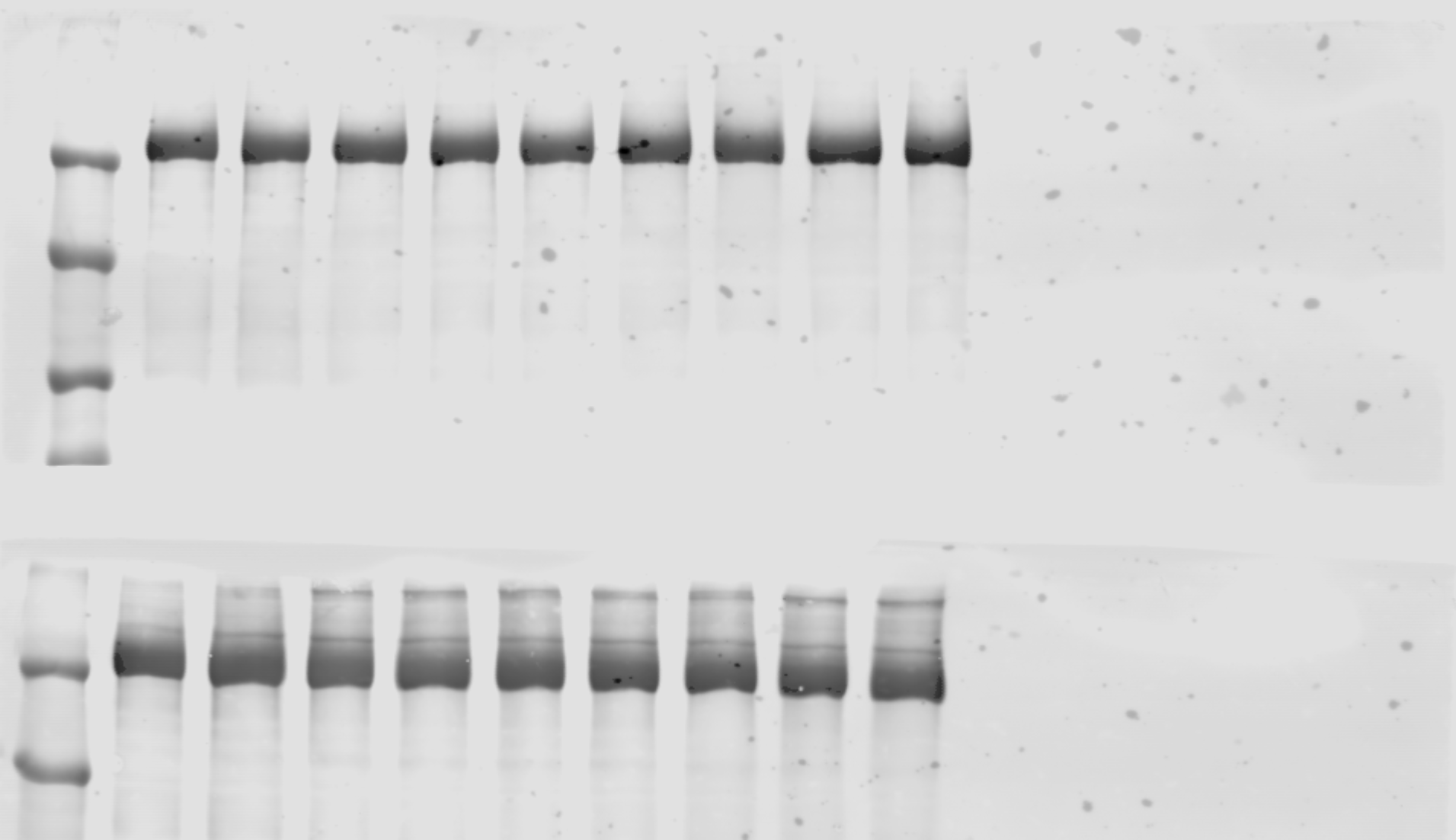

Supplement: Supplementary file 4 — Source Data [file 41467_2022_30060_MOESM4_ESM.zip › source_data/Figure 4/Fig4f_2020-09-18_HA4-FAS_ph7.tif]

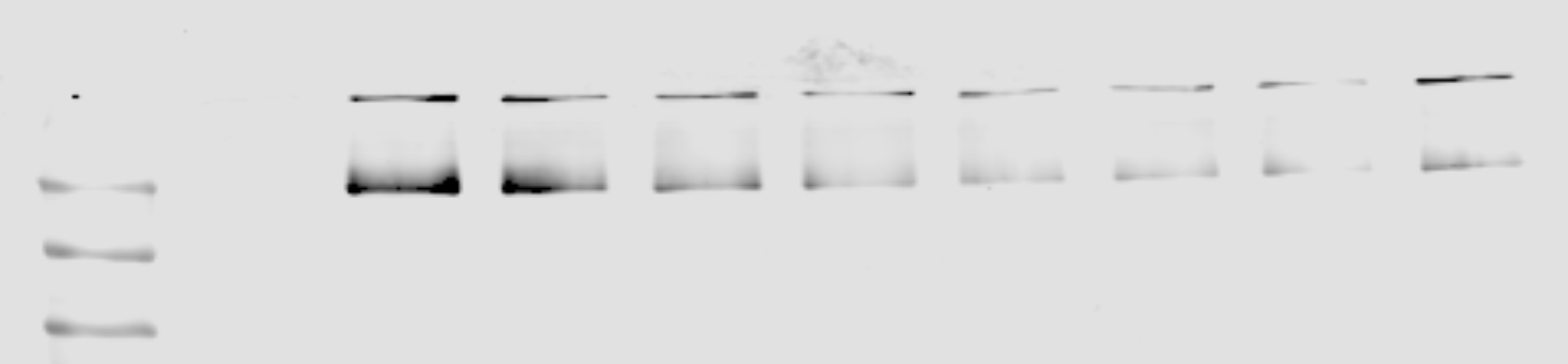

Supplement: Supplementary file 4 — Source Data [file 41467_2022_30060_MOESM4_ESM.zip › source_data/Figure 4/Fig4b_HMG_2016-08-05_HMG pre load_Mal Comp_HMG signal.tif]

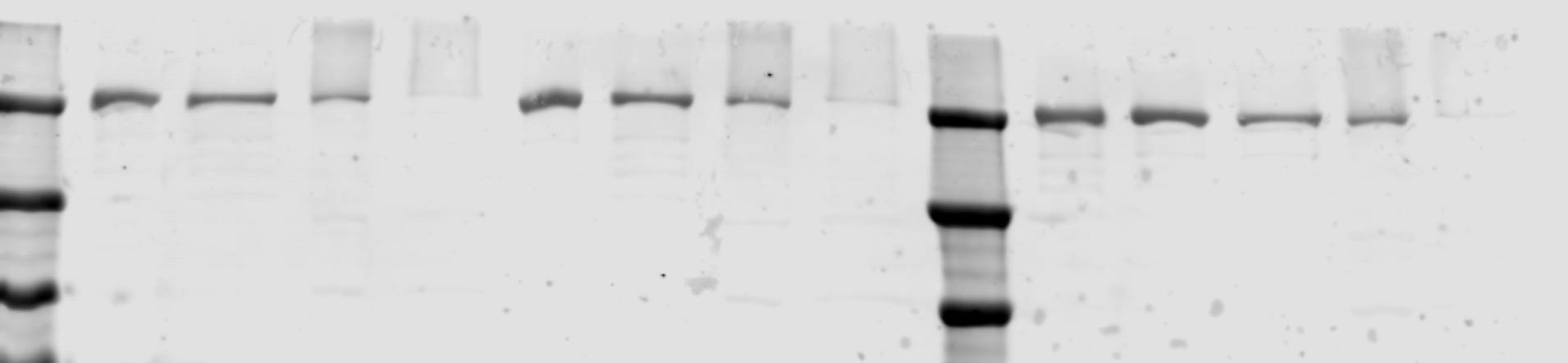

Supplement: Supplementary file 4 — Source Data [file 41467_2022_30060_MOESM4_ESM.zip › source_data/Figure 4/Fig4e_2020-09-19_HeatRed3_FAS.tif]

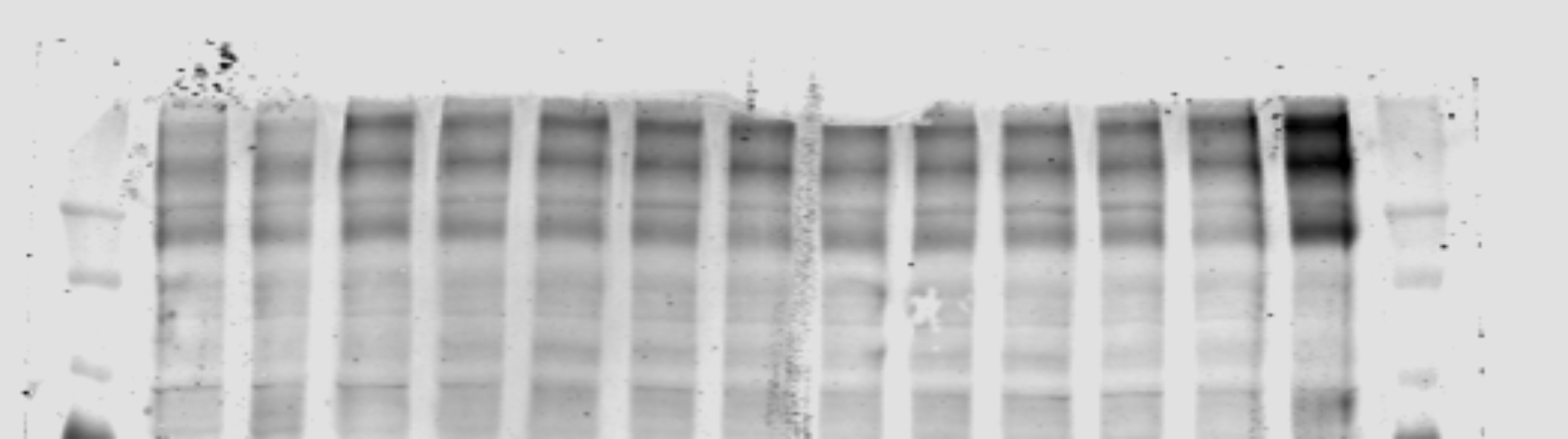

Supplement: Supplementary file 4 — Source Data [file 41467_2022_30060_MOESM4_ESM.zip › source_data/Figure 4/Fig4c_2017-04-26_Statin short course 3_HMGCR.tif]

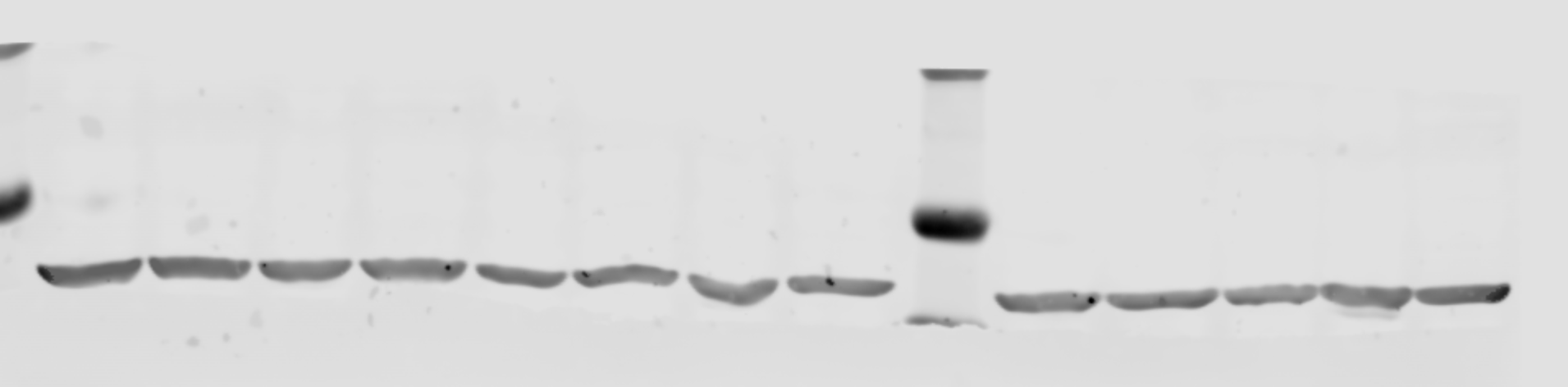

Supplement: Supplementary file 4 — Source Data [file 41467_2022_30060_MOESM4_ESM.zip › source_data/Figure 4/Fig4e_2020-09-18_HeatRed3_bactin.tif]

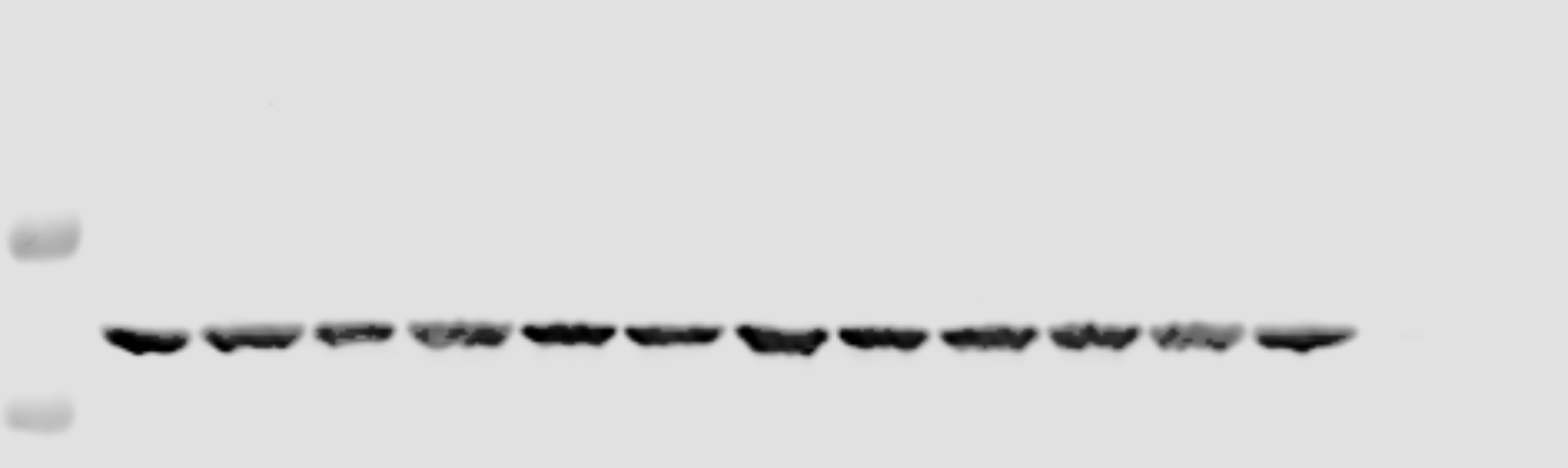

Supplement: Supplementary file 4 — Source Data [file 41467_2022_30060_MOESM4_ESM.zip › source_data/Figure 3/Fig3c_2017-06-15_Hymeglucin dose curve 2 siv_b actin.tif]

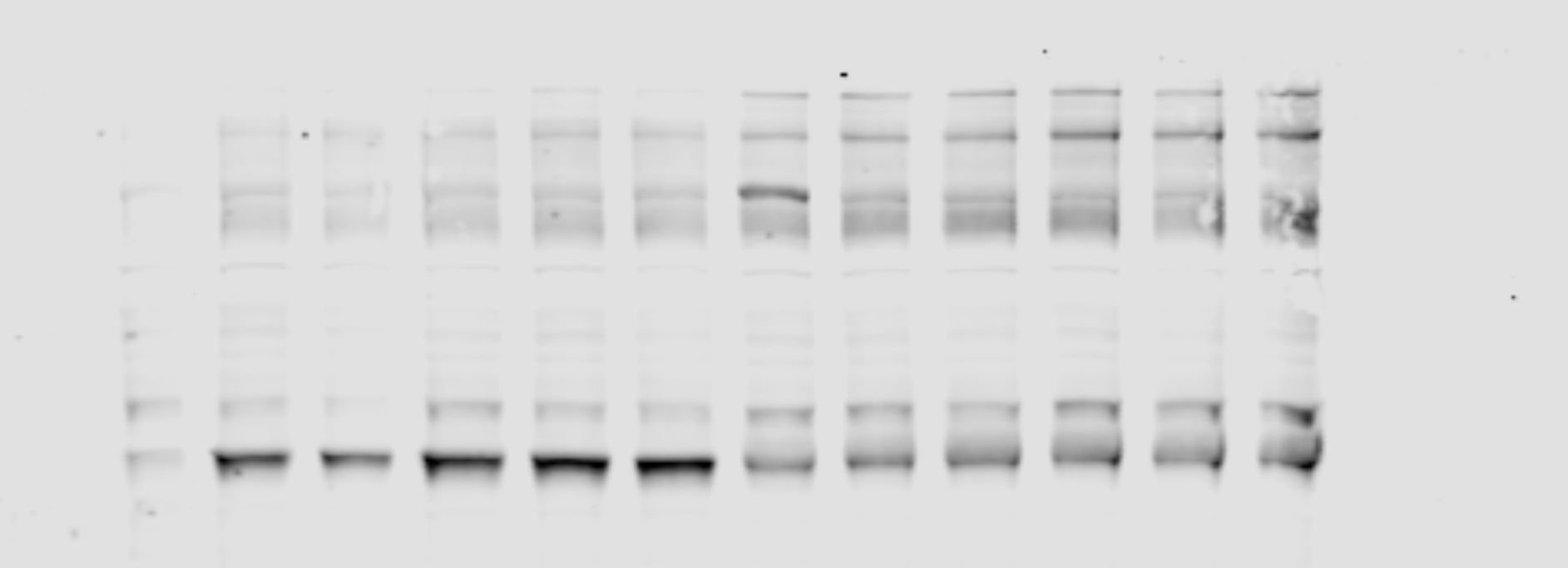

Supplement: Supplementary file 4 — Source Data [file 41467_2022_30060_MOESM4_ESM.zip › source_data/Figure 3/Fig3c_HMGCR_2017-06-16_Hymeglucin dose curve 2 siv_HMGCR.tif]

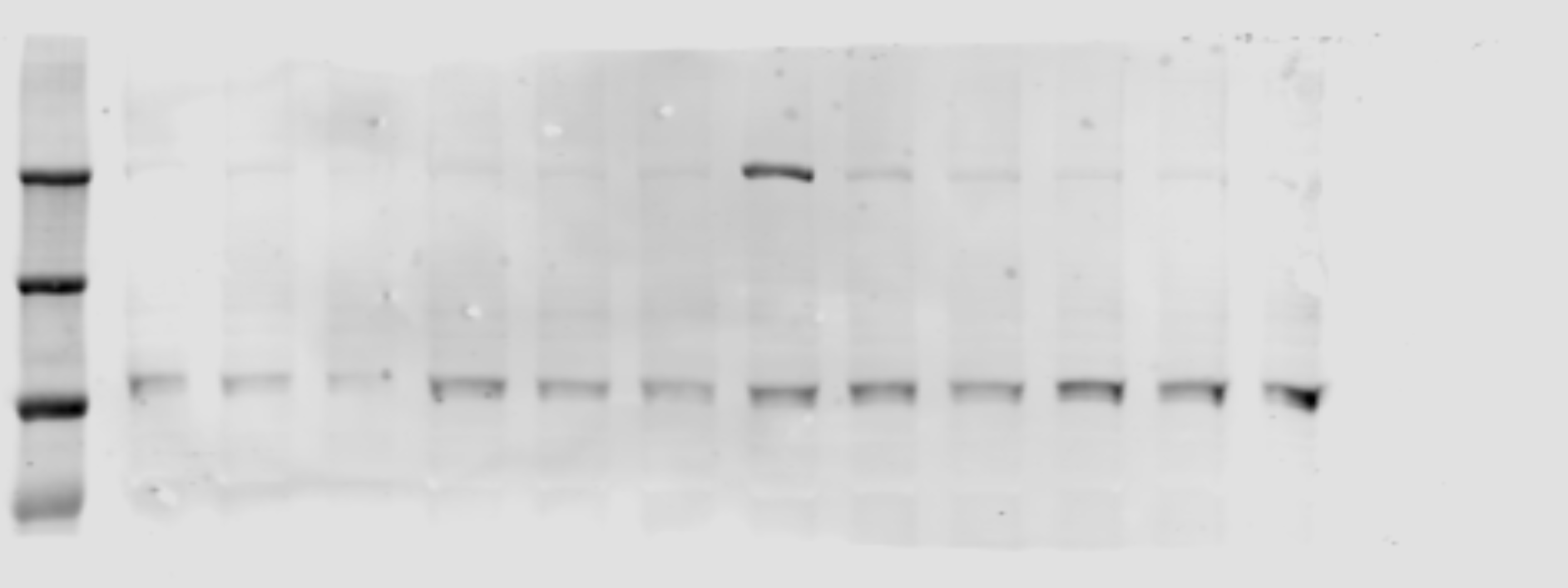

Supplement: Supplementary file 4 — Source Data [file 41467_2022_30060_MOESM4_ESM.zip › source_data/Figure 3/Fig3c_HMGylation_2017-06-15_Hymeglucin dose curve 2 siv_HMG.tif]

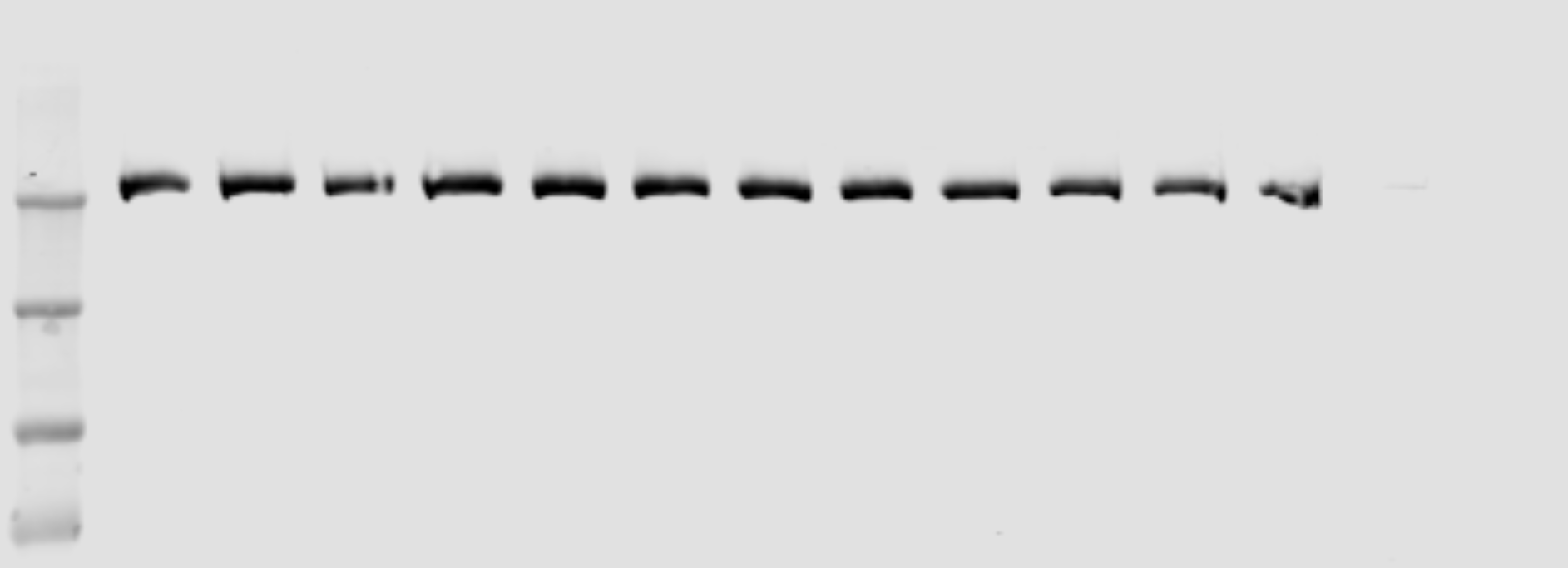

Supplement: Supplementary file 4 — Source Data [file 41467_2022_30060_MOESM4_ESM.zip › source_data/Figure 3/Fig3c_FAS_2017-06-16_Hymeglucin dose curve 2 siv_FAS.tif]

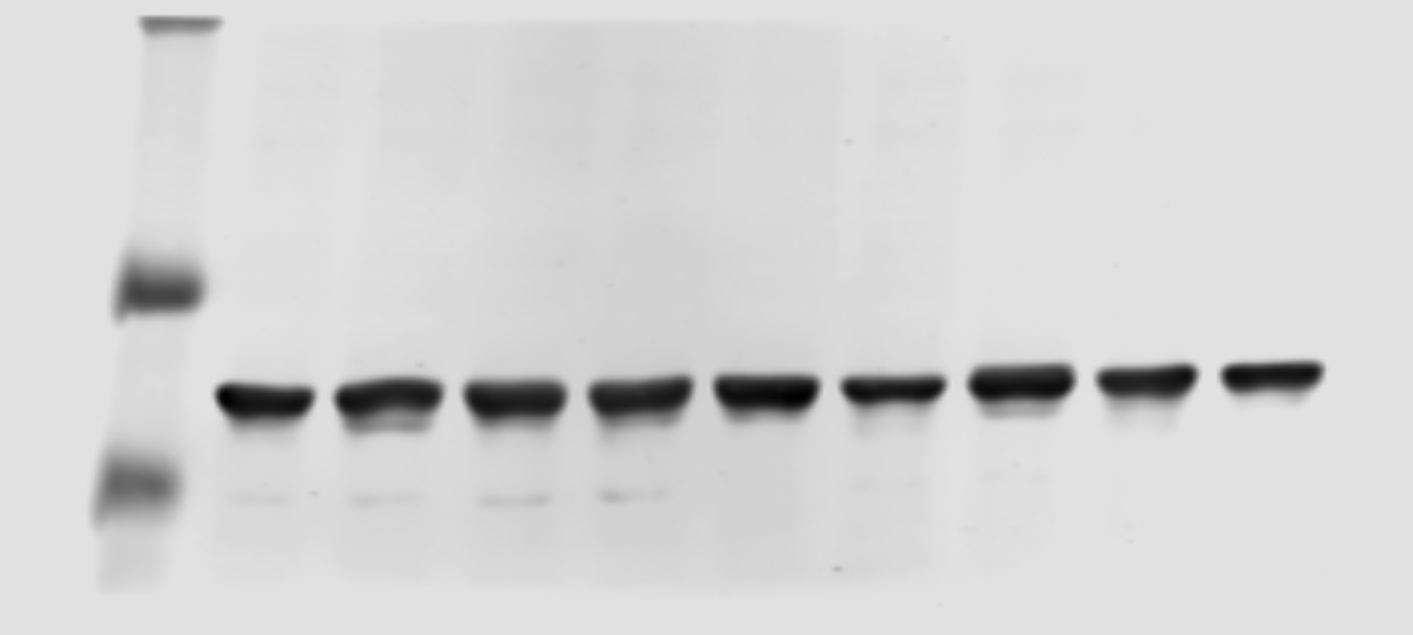

Supplement: Supplementary file 4 — Source Data [file 41467_2022_30060_MOESM4_ESM.zip › source_data/Figure 2/Fig2c_betaactin_2018-03-01_HMGNAC3Lysate_bactin.tif]

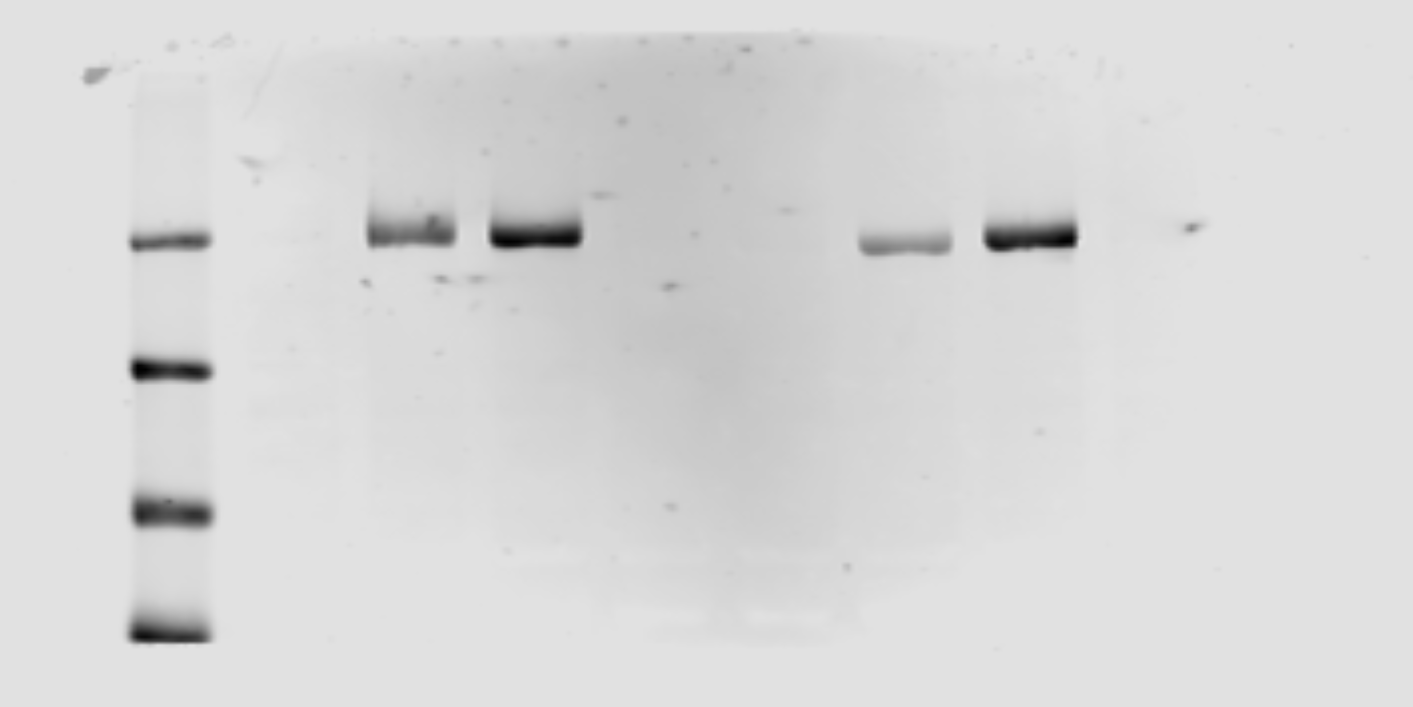

Supplement: Supplementary file 4 — Source Data [file 41467_2022_30060_MOESM4_ESM.zip › source_data/Figure 2/Fig2c_HMGylation_2018-03-01_HMGNAC3Lysate_HMG.tif]

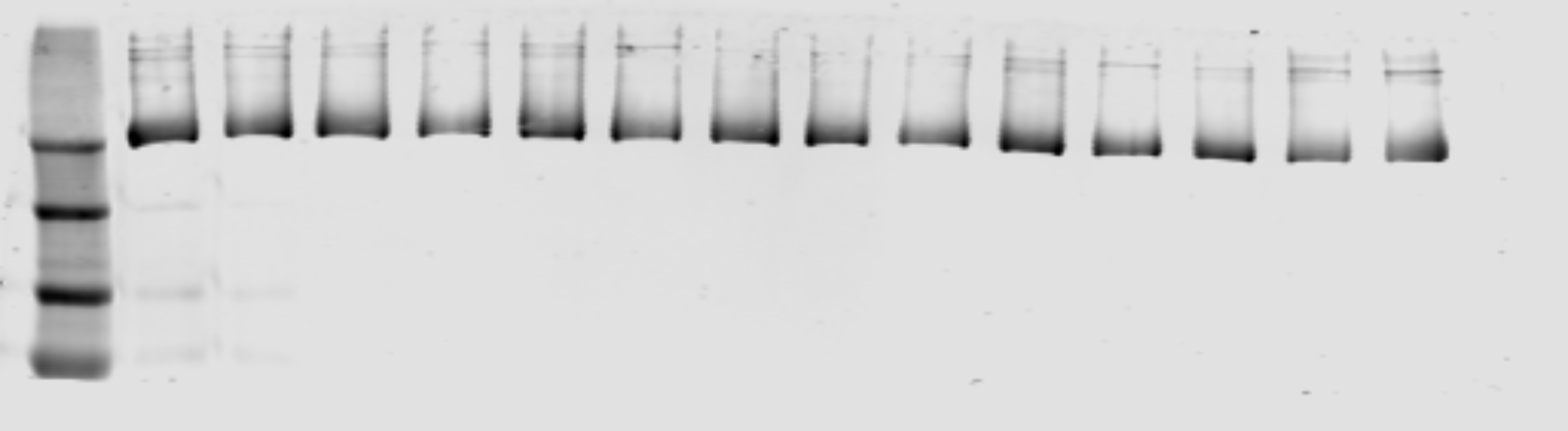

Supplement: Supplementary file 4 — Source Data [file 41467_2022_30060_MOESM4_ESM.zip › source_data/Figure 2/Fig2a_FAS_2016-04-23_HMG time FAS.tif]

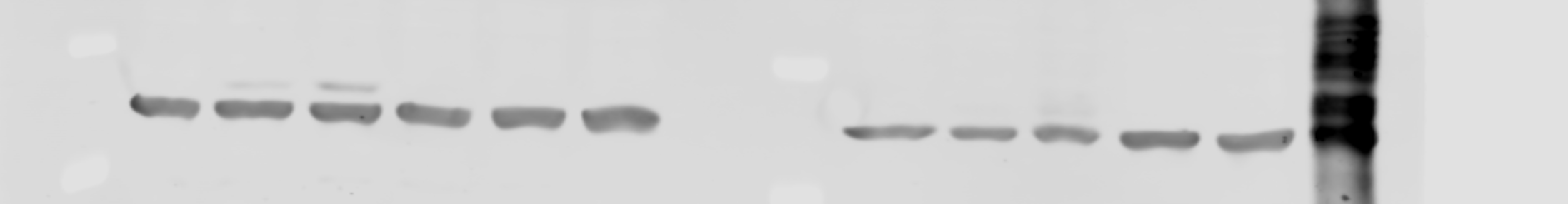

Supplement: Supplementary file 4 — Source Data [file 41467_2022_30060_MOESM4_ESM.zip › source_data/Figure 2/Fig2b_betaactin_denaturation2_bactin.tif]

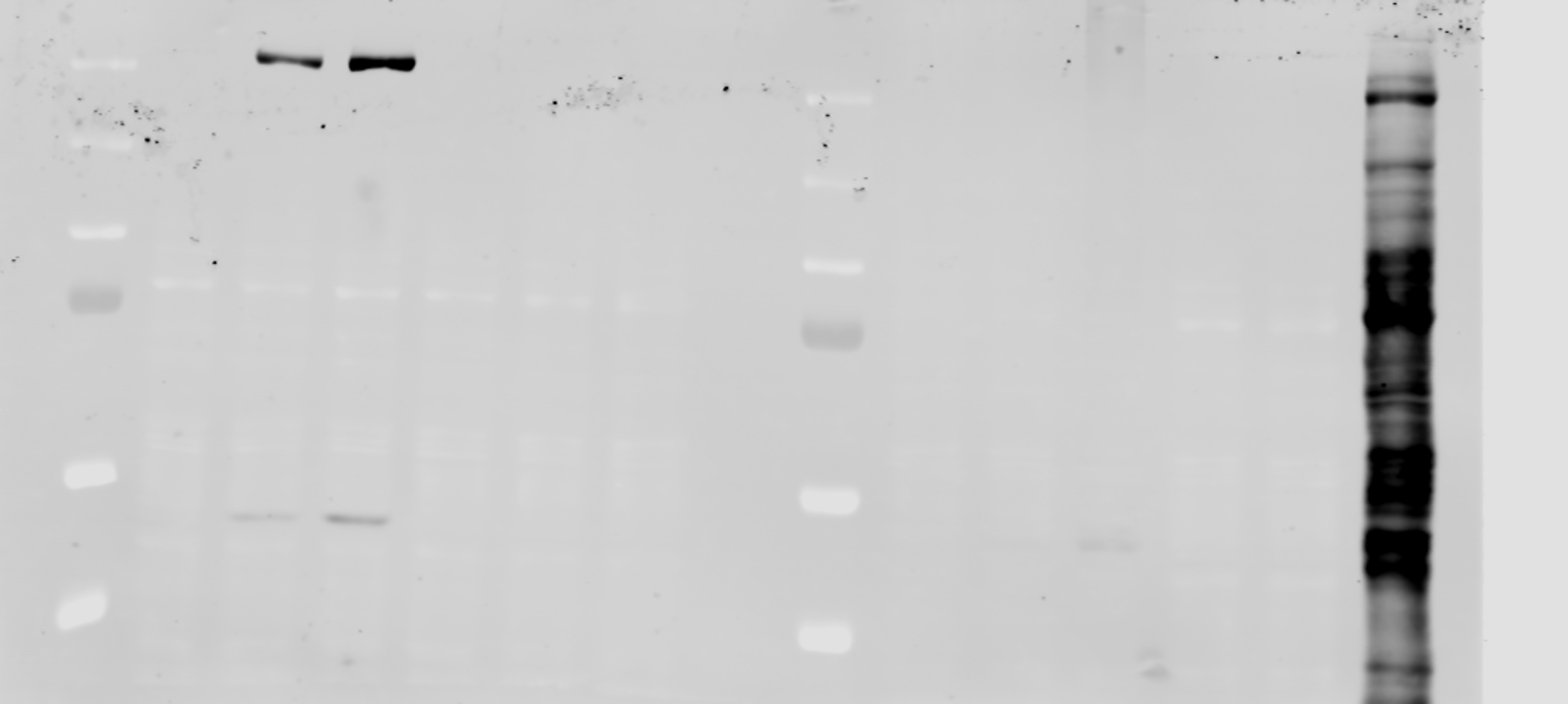

Supplement: Supplementary file 4 — Source Data [file 41467_2022_30060_MOESM4_ESM.zip › source_data/Figure 2/Fig2b_HMGylation_denaturation2_HMG.tif]

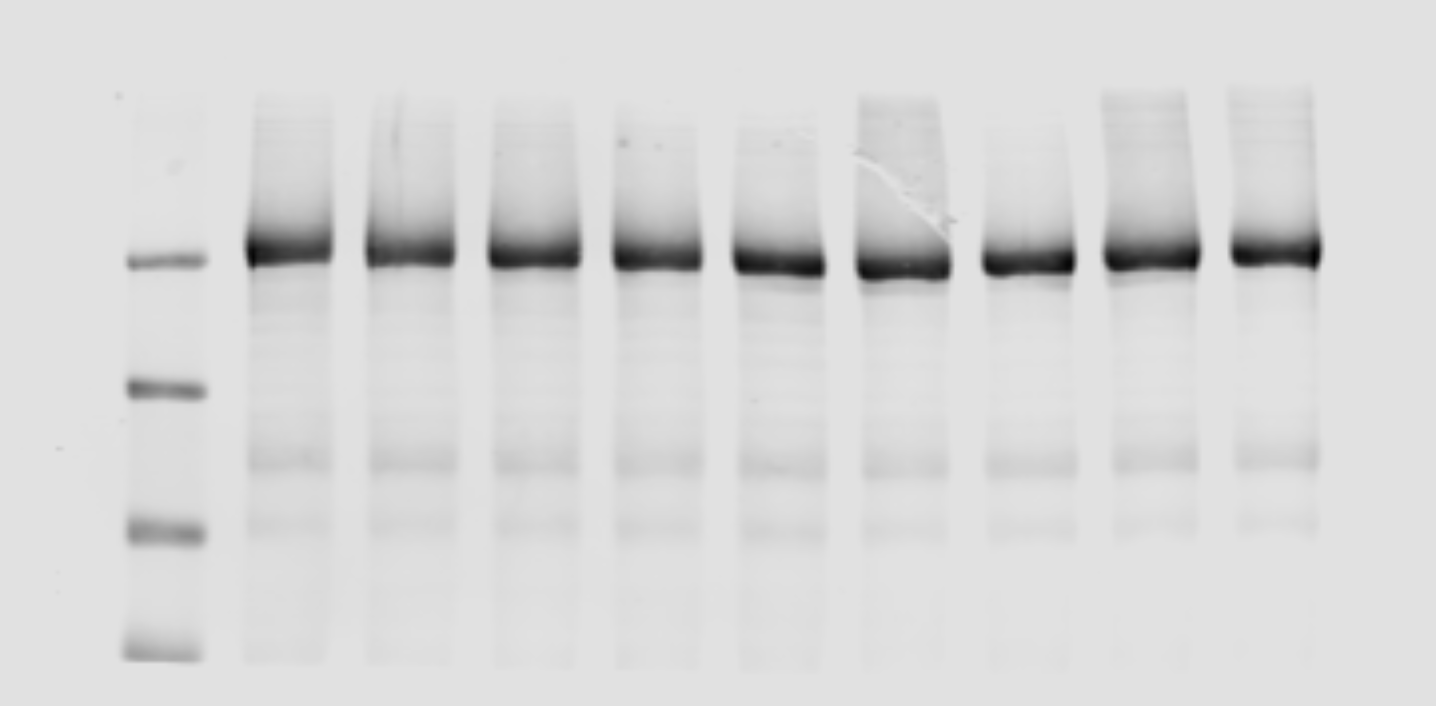

Supplement: Supplementary file 4 — Source Data [file 41467_2022_30060_MOESM4_ESM.zip › source_data/Figure 2/Fig2c_FAS_2018-03-11_HMGNAC3Lysate_FAS.tif]

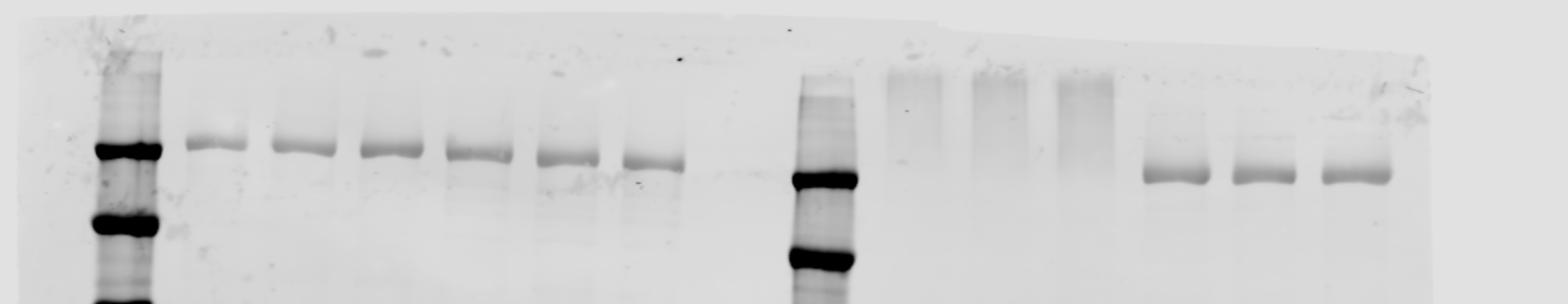

Supplement: Supplementary file 4 — Source Data [file 41467_2022_30060_MOESM4_ESM.zip › source_data/Figure 2/Fig2b_FAS_denaturation2_FAS.tif]

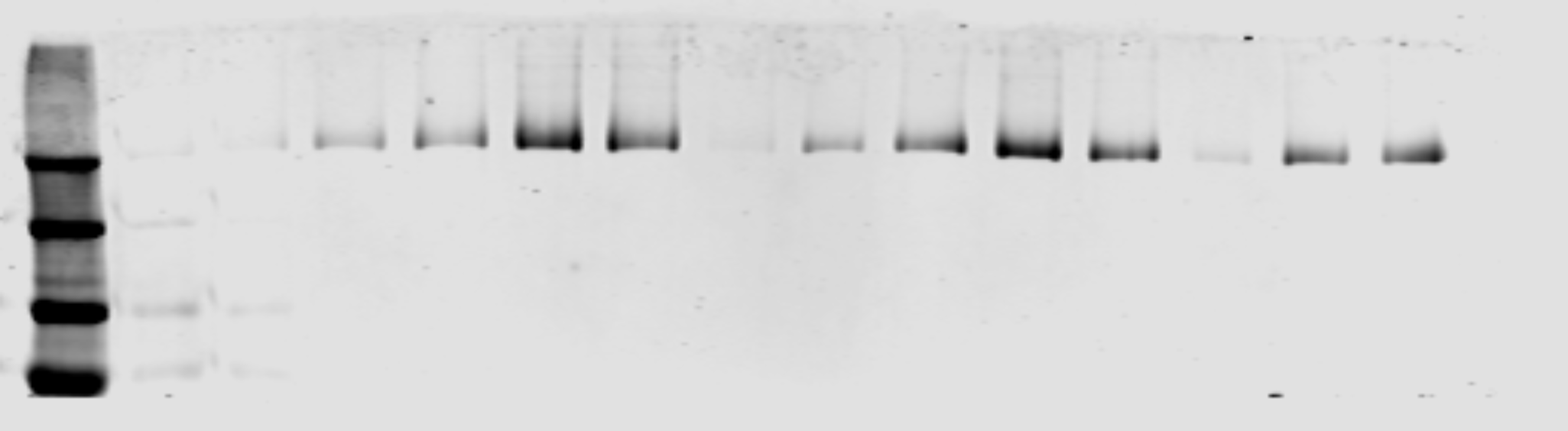

Supplement: Supplementary file 4 — Source Data [file 41467_2022_30060_MOESM4_ESM.zip › source_data/Figure 2/Fig2a_HMGylation_2016-04-22_HMG loading time course.tif]
